# Size-Controlled and Surface-Modified LDH Nanofillers Enable Multifunctional Transparent PMMA Nanocomposites with Enhanced Thermal Stability and UV Shielding

**DOI:** 10.3390/ijms272110037

**Published:** 2026-11-09

**Authors:** Tsung-Yen Tsai, Pin-Yu Chiang, Shao-An Yeh, Reza Bahrami, Hung-Jue Sue

**Affiliations:** 1 Department of Chemistry, Chung Yuan Christian University, Chung-Li 32023, Taiwan; pinyujiang@gmail.com (P.-Y.C.); samyeh9009@gmail.com (S.-A.Y.); 2 Center for Nanotechnology, Chung Yuan Christian University, Chung-Li 32023, Taiwan; 3 R&D Center for Membrane Technology, Chung Yuan Christian University, Chung-Li 32023, Taiwan; 4 Department of Materials Science and Engineering, Polymer Technology Center, Texas A&M University, College Station, TX 77843, USA; reza.bahrami@tamu.edu (R.B.); hjsue@tamu.edu (H.-J.S.)

**Keywords:** PMMA, LDH, nanocomposites, emulsion polymerization, thermal stability, UV shielding

## Abstract

Poly(methyl methacrylate) (PMMA)/layered double hydroxide (LDH) nanocomposites were prepared via emulsion polymerization to investigate the effects of LDH platelet size and surface modification on nanocomposite structure and performance. Size-controlled MgAl-LDH and ZnAl-LDH were synthesized by aqueous co-precipitation, organically modified with various interlayer agents, and incorporated into the PMMA matrix. Structural, morphological, thermal, mechanical, and optical properties were systematically characterized using X-ray diffraction (XRD), Fourier-transform infrared spectroscopy (FT-IR), thermogravimetric analysis (TGA), dynamic mechanical analysis (DMA), scanning and transmission electron microscopy (SEM/TEM), polarized optical microscopy (POM), and UV-visible spectroscopy. The results demonstrated that nanoscale LDH dispersion could be achieved at low filler loadings (1–3 phr), whereas increasing the LDH content to 5 phr promoted significant platelet aggregation. Notably, a high degree of LDH intercalation did not necessarily translate into improved dispersion. Despite exhibiting the greatest interlayer expansion, CDS-modified LDHs showed inferior dispersion and thermal performance, indicating that interfacial compatibility rather than interlayer spacing is the primary factor governing nanocomposite morphology and properties. The optimized PMMA/LDH nanocomposites retained over 85% visible-light transmittance while exhibiting a 27% enhancement in UV-shielding efficiency, improved thermal stability as evidenced by an increased decomposition temperature (T_5d_), and a higher storage modulus relative to neat PMMA. These findings demonstrate that the synergistic control of LDH platelet size and surface chemistry provides an effective strategy for developing highly dispersed PMMA nanocomposites with simultaneously enhanced optical, thermal, and mechanical properties.

## 1. Introduction

Poly (methyl methacrylate) (PMMA) is a widely used transparent thermoplastic valued for its high optical clarity, excellent processability, and non-toxicity. Since the earliest synthesis of methacrylate esters by Caspary and Tollens [1] in 1873 and the first successful PMMA polymerization by Wilhelm Rudolph Fitting [2] in 1877, PMMA has evolved into an essential material across optical devices, construction materials, automotive components, biomedical products, and electronic displays. Industrial-scale development accelerated in the early 20th century when Dr. Otto Röhm [3,4] elucidated the polymerization behavior of methacrylate monomers, enabling Imperial Chemical Industries (ICI) to commercialize PMMA sheets in 1936. Today, global PMMA consumption exceeds 2.3 million tons annually, driven by electronics (33%), automotive (24%), medical (18%), and construction sectors (16%).

Despite its broad applicability, PMMA suffers from intrinsic limitations, including insufficient thermal resistance, inadequate mechanical strength, UV-induced yellowing, surface degradation, and poor long-term durability under outdoor exposure. Numerous polymer nanocomposite systems have been explored to address these issues, yet achieving simultaneous improvements in thermal, mechanical, and optical properties remains challenging. Early breakthroughs in the late 1990s by Emmanuel P. Giannelis [5,6] demonstrated that incorporating only 2–5 wt.% layered silicates could raise PMMA’s decomposition temperature by 40–50 °C while maintaining 85–90% transparency. In the early 2000s, Charles A. Wilkie [7,8] became the most prolific contributor in PMMA/clay flame-retardant systems, showing that organically modified clays could delay PMMA thermal degradation by ~50 °C and form protective char–clay layers. Complementary reviews by Alexandre and Dubois [9] further confirmed that organically modified montmorillonite (MMT) enhances PMMA’s mechanical strength by 20–50% and reduces gas permeability by 30–70%.

Carbon-based nanofillers such as CNTs and graphene provide the strongest mechanical reinforcement among PMMA nanocomposites; for example, Raina Aman Qazi [10] (2021) achieved the highest thermal stability enhancement to date, with a remarkable 127 °C increase in T_5d_ using functionalized multi-walled carbon nanotubes. However, their intrinsic broadband optical absorption significantly reduces visible-light transmittance, limiting their suitability for transparent applications.

Layered double hydroxides (LDHs) offer a promising alternative due to their tunable layered structure, flexible ionic composition, and versatile interlayer organic modification capability. First discovered in natural hydrotalcite by Hochstetter [11] in 1842 and structurally confirmed by Taylor and Allmann [12] in 1967, LDHs have attracted substantial research interest, with more than 30,832 publications between 2015 and 2024. PMMA/LDH nanocomposites were first reported by Wang G.-A [13] in 2006, establishing the foundation for subsequent studies. The most active research group in the 2010s, led by José Costa de Macedo Neto and Telma Nogueira [14] (UFPE, Brazil), published 10 representative papers on PMMA/LDH systems. Recent work by Oliveira [15] (2025) demonstrated that even 0.25 wt.% LDH can significantly improve PMMA’s resistance to γ- radiation, mechanical degradation, and surface roughening.

Despite these advances, existing PMMA/LDH studies remain fragmented, with most investigations focusing on individual LDH compositions, specific intercalating agents, or single-property enhancements. Systematic studies that simultaneously evaluate the coupled effects of LDH platelet size, interlayer organic modifiers, and dispersion quality on PMMA transparency, thermal stability, mechanical performance, and UV-shielding behavior remain scarce. Therefore, a comprehensive structure-property investigation is required to establish rational design principles for multifunctional transparent PMMA/LDH nanocomposites. Moreover, conventional inorganic fillers such as TiO_2_ and SiO_2_, although effective in enhancing hardness or UV protection, frequently compromise transparency or induce photocatalytic degradation. In contrast, LDHs possess intrinsically transparent layered structures, tunable metal compositions, and exchangeable interlayer anions, allowing simultaneous enhancement of thermal stability and UV shielding while preserving optical transparency.

Overall, this work establishes LDHs as a key enabler for developing high-performance transparent polymer nanocomposites. The demonstrated strategy provides a scalable and reproducible pathway for producing multifunctional PMMA-based materials, opening new opportunities for applications in optical engineering, energy technologies, and biomedical devices.

## 2. Results and Discussion

### 2.1. Characterization of Inorganic Layered Materials

**Figure 1:**
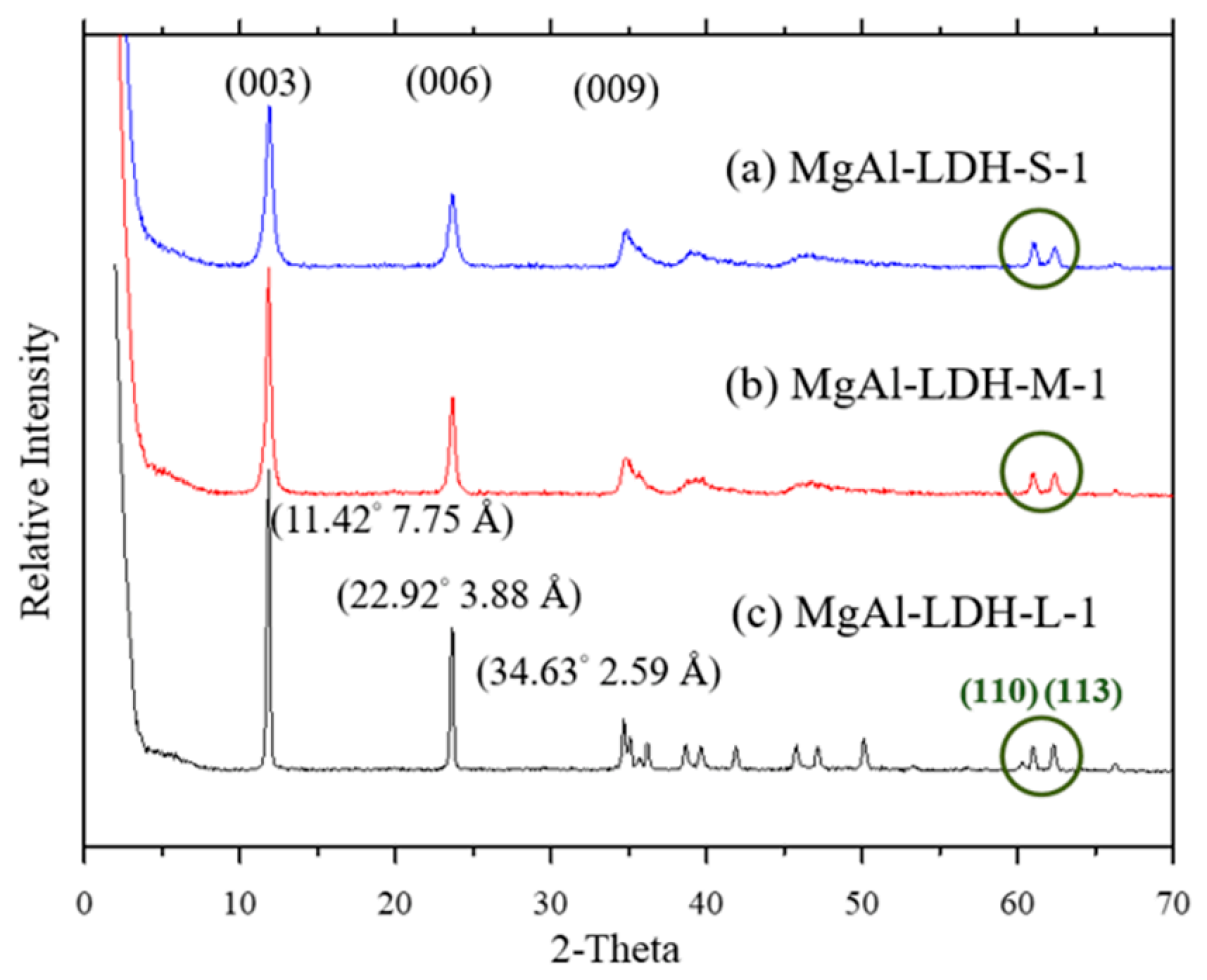
XRD patterns of MgAl-LDH after 1 min of nucleation at (a) 100 °C, 6 h; (b) 100 °C, 15 h; and (c) 150 °C, 24 h.

**Figure 2:**
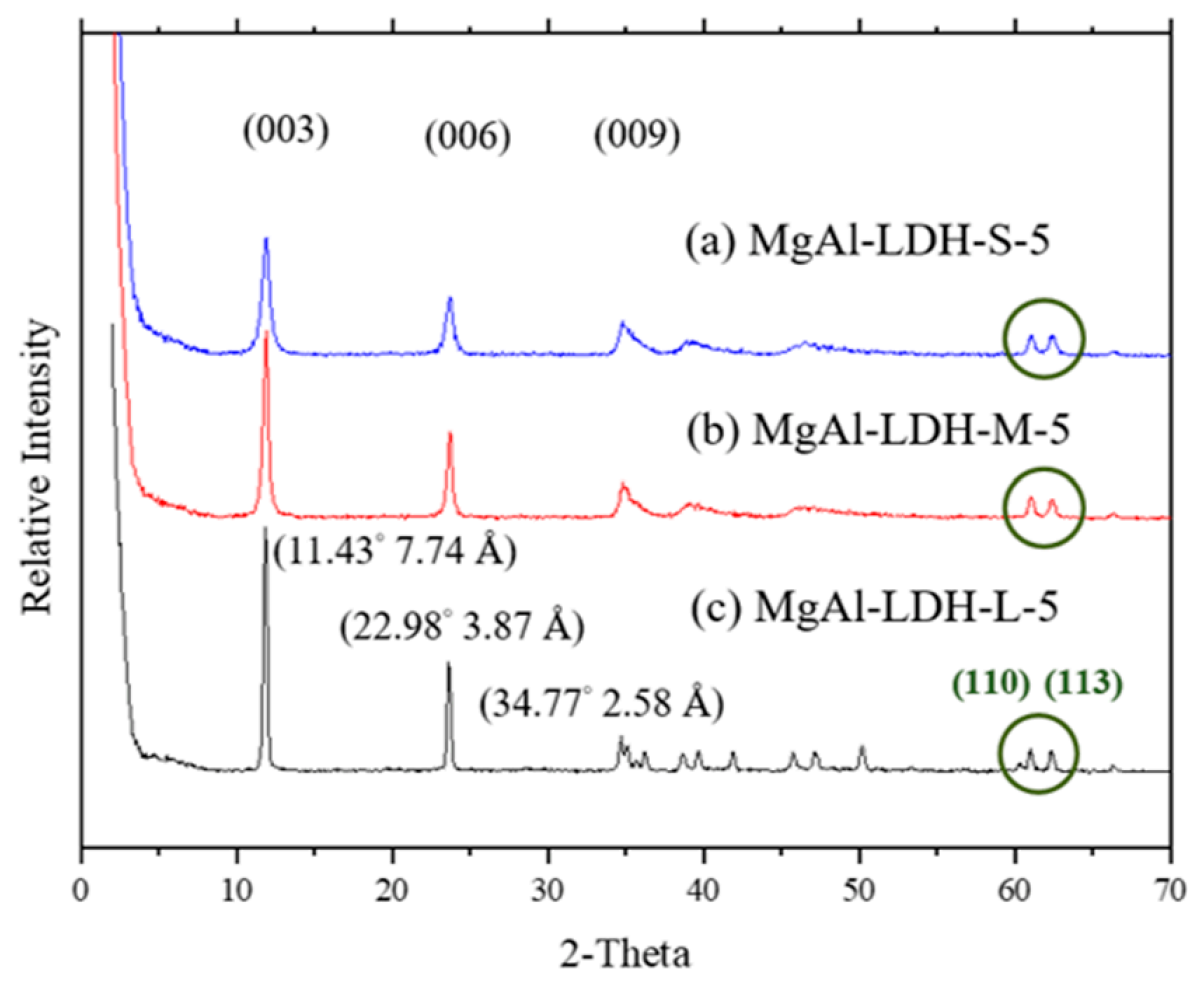
XRD patterns of MgAl-LDH after 5 min of nucleation at (a) 100 °C, 6 h; (b) 100 °C, 15 h; and (c) 150 °C, 24 h.

Figure 1 and Figure 2 display the typical diffraction features of all six samples, showing characteristic LDH reflections at low angles corresponding to the (003), (006), and (009) planes. The MgAl-LDH sample obtained after 1 min of nucleation already exhibits identifiable LDH reflections, indicating the layered structure forms rapidly at the earliest stage of synthesis and a pronounced increase in crystallinity under all hydrothermal conditions (100 °C/6 h, 100 °C/15 h, 150 °C/24 h). The basal reflections become sharper and more intense, reflecting rapid structural refinement. The sample at 150 °C/24 h displays the narrowest peaks, indicating highly ordered stacking, whereas the 100 °C/6 h sample retains broader peaks, suggesting restricted structural reorganization at lower thermal input. After 5 min of nucleation, MgAl-LDH samples show that although the basal peaks are present, their broader shapes and lower intensities suggest limited long-range ordering and incomplete layer stacking.

The observed platelet-size evolution is consistent with the classical LaMer nucleation-growth model [16]. Short nucleation times generate fewer nuclei, allowing greater crystal growth and resulting in larger, more crystalline LDH platelets. Conversely, prolonged nucleation increases the number of nuclei and limits individual crystal growth, producing smaller platelets. According to Bragg’s law, the basal spacings are calculated as 7.74, 3.87, and 2.58 Å. The presence of (110) and (113) reflections at 2θ = 60–65° further confirms the formation of a well-ordered layered structure. A clear increase in crystallinity is observed from S to M to L samples, with LDH-L exhibiting the sharpest diffraction peaks, indicative of improved crystal growth and structural ordering.

Overall, the XRD results not only verify the successful synthesis of LDHs but also demonstrate that platelet size control significantly improves crystallinity, thereby establishing a more robust structural basis for enhanced dispersion and subsequent performance in composite systems.

**Figure 3:**
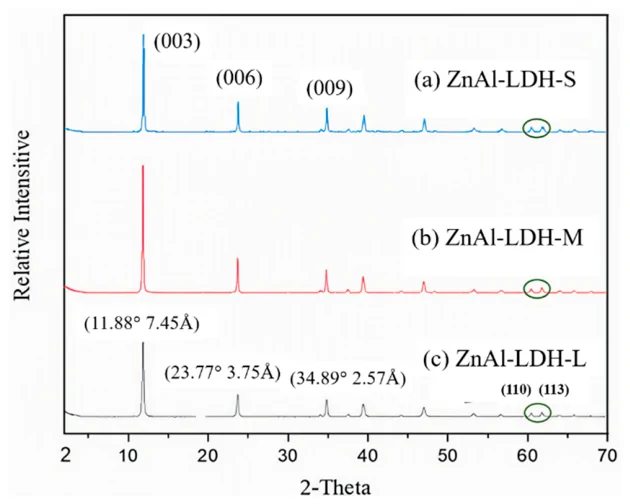
XRD patterns of ZnAl-LDH after 10 min of nucleation at 150 °C after (a) 6 h; (b) 12 h; and (c) 24 h.

The platelet growth behavior of ZnAl-LDH was examined under a nucleation time of 10 min followed by hydrothermal treatment at 150 °C. As shown in Figure 3, the LDH phase is already established within the first 10 min, indicating rapid nucleation like the MgAl system. However, ZnAl-LDH exhibits a more pronounced sensitivity to hydrothermal reaction time. The sample treated for 6 h displays moderately sharp and intense reflections, suggesting that the shortened reaction duration limits crystal growth and results in partially developed layer stacking. Extending the reaction time to 12 h produces the most intense and well-defined diffraction peaks, reflecting optimal crystal growth and highly ordered lattice stacking. In contrast, further prolongation to 24 h leads to a noticeable decrease in peak intensity and broadening of reflections, implying that excessive hydrothermal exposure induces structural degradation and defect formation on the ZnAl-LDH surface, thereby reducing overall crystallinity. Combining the results shown in Figure 1, Figure 2 and Figure 3, it is evident that LDH nucleation occurs rapidly (within 1–10 min), but the degree of crystallinity is governed by both hydrothermal severity and platelet size. Larger platelets contain more stable and fewer nucleation centers, enabling faster and more complete structural development. Higher temperatures and longer reaction durations further enhance peak resolution, while smaller platelets remain limited by surface disorder and insufficient nucleation density.

These structural advantages provide a robust foundation for improved dispersion, interfacial compatibility, and functional performance in subsequent composite or catalytic applications.

**Figure 4:**
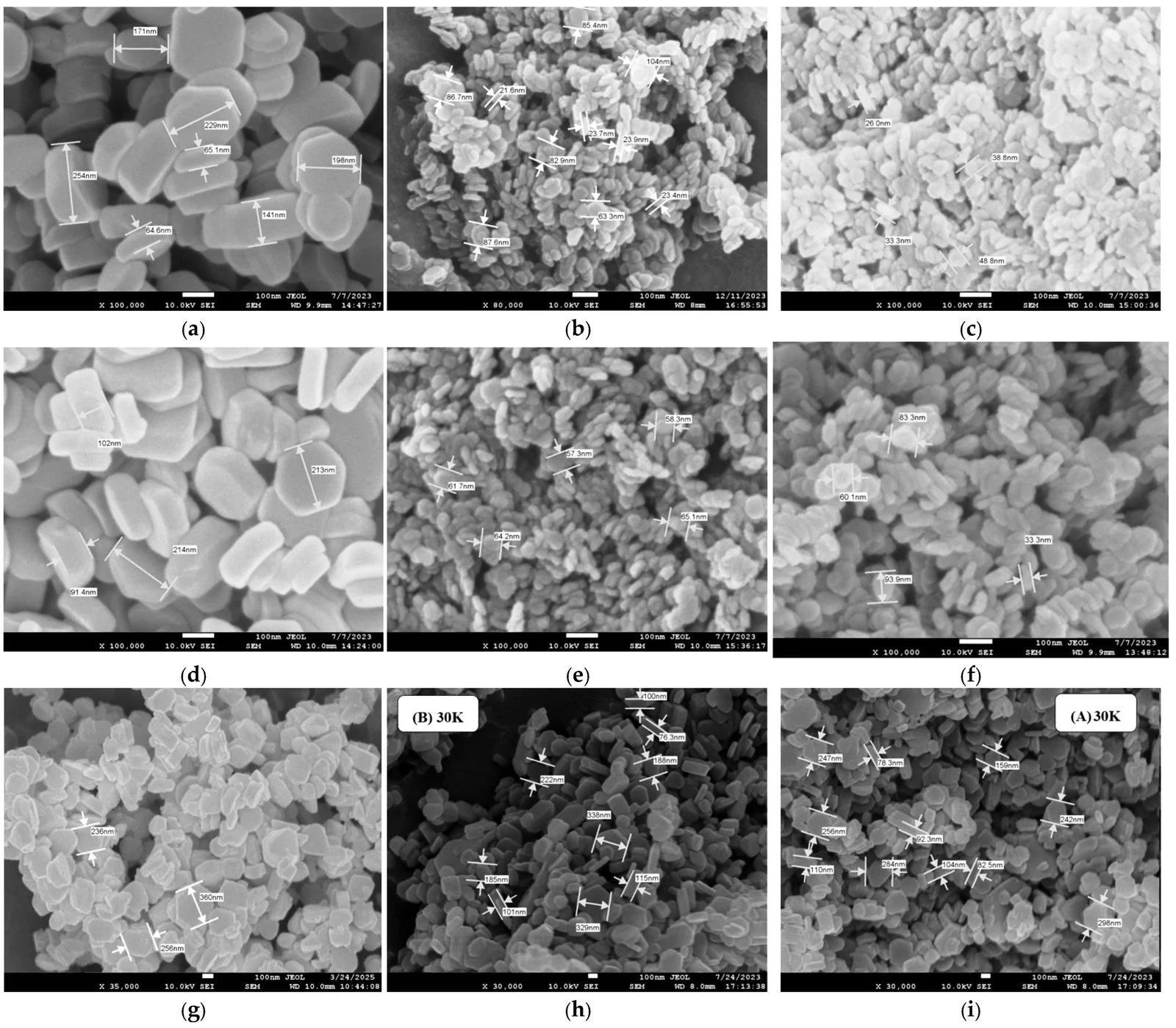
SEM images of LDH synthesized under different conditions: (**a**) MgAl-LDH-L-1, (**b**) MgAl-LDH-M-1, (**c**) MgAl-LDH-S-1, (**d**) MgAl-LDH-L-5, (**e**) MgAl-LDH-M-5, (**f**) MgAl-LDH-S-5, (**g**) ZnAl-LDH-L-10, (**h**) ZnAl-LDH-M-10, and (**i**) ZnAl-LDH-S-10. The scale bar corresponds to 100 nm.

XRD and SEM analyses jointly verify a well-defined layered structure and a reproducible lateral-size hierarchy across the MgAl-LDH and ZnAl-LDH series. SEM (Figure 4) shows that L-, M-, and S-type platelets exhibit progressively reduced lateral dimensions consistent with DLS results (shown in Table 1), with large platelets displaying smooth hexagonal morphologies and smaller ones showing increasing edge irregularity and defect density. This morphological trend aligns with the XRD observations, where L-type samples present sharper and more intense basal reflections, indicating higher crystallinity and more coherent lamellar stacking. Together, these results demonstrate that controlled hydrothermal conditions reliably tune platelet size and structural order, providing a solid basis for subsequent evaluation of size-dependent dispersion and property enhancement in PMMA nanocomposites.

The DLS platelet sizes are generally larger than the SEM-derived lateral dimensions because DLS measures the hydrodynamic diameter of dispersed platelets and small aggregates in suspension, whereas SEM characterizes the dimensions of individual dried platelets.

According to previous studies [17,18,19,20], the chemical compositions of MgAl-LDH and ZnAl-LDH can be derived from elemental analysis and ICP-MS measurements. The MgAl-LDH sample is represented by the formula [Mg_2_Al(OH)_6_](NO_3_)_0.2_(CO_3_)_0.4_•n H_2_O, corresponding to an anion exchange capacity (AEC) of 3.80 meq g^−1^ of LDH.

In contrast, the ZnAl-LDH sample is described by the formula [Zn_2_Al(OH)_6_](NO_3_)_0.1_(CO_3_)_0.45_•n H_2_O, with a lower anion exchange capacity (AEC) of 2.85 meq g^−1^ of LDH.

These compositional differences reflect the inherently higher layer charge density of MgAl-LDH relative to ZnAl-LDH, which directly influences their anion-exchange behavior and intercalation capacity.

### 2.2. Intercalation Behavior and Morphology of Modified LDHs

**Figure 5:**
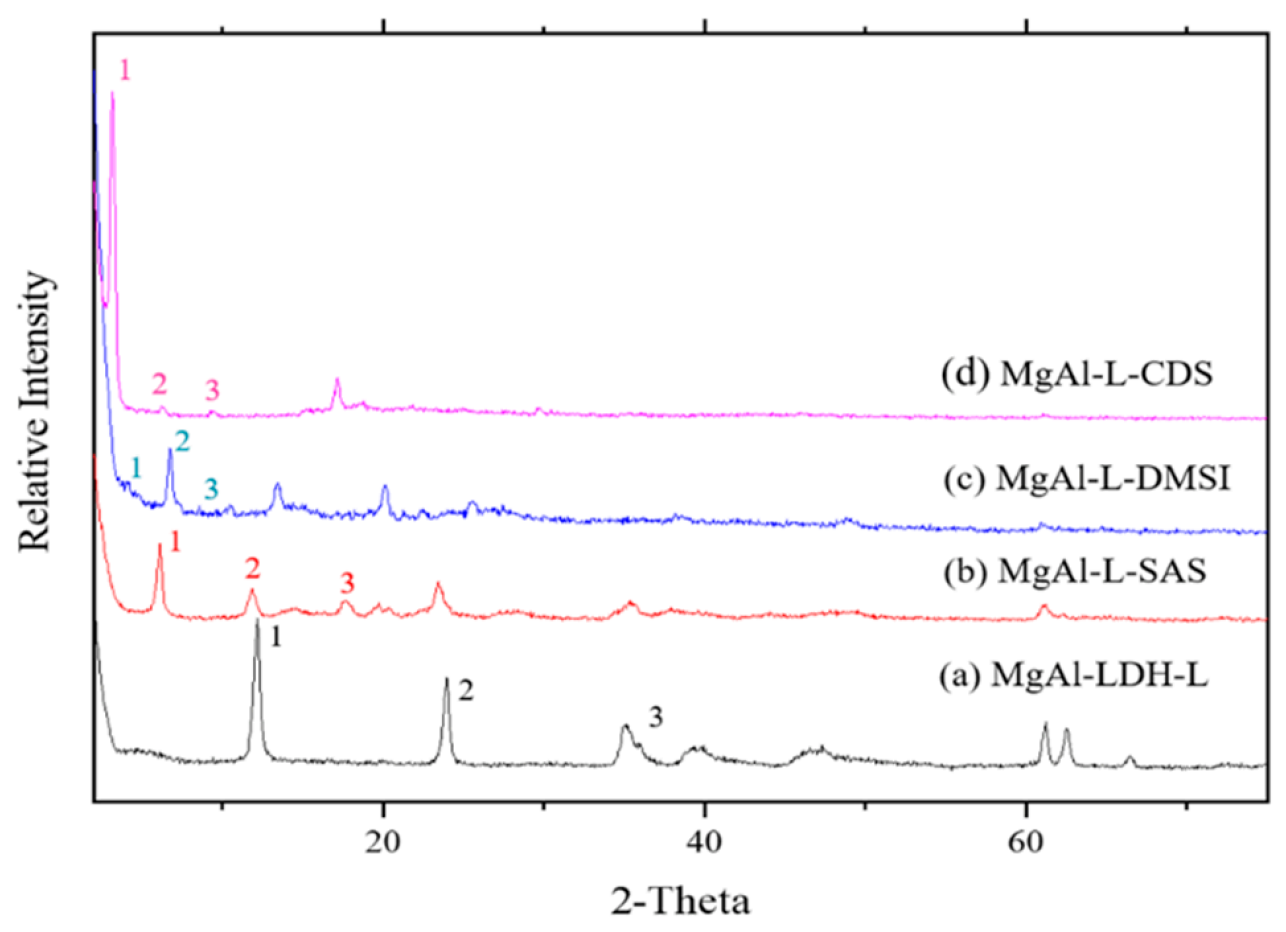
XRD patterns of MgAl-LDH with different modifiers: (a) MgAl-LDH-L, (b) MgAl-L-SAS, (c) MgAl-L-DMSI, and (d) MgAl-L-CDS. Peaks 1, 2, and 3 are assigned to the (003), (006), and (009) reflections, respectively.

**Figure 6:**
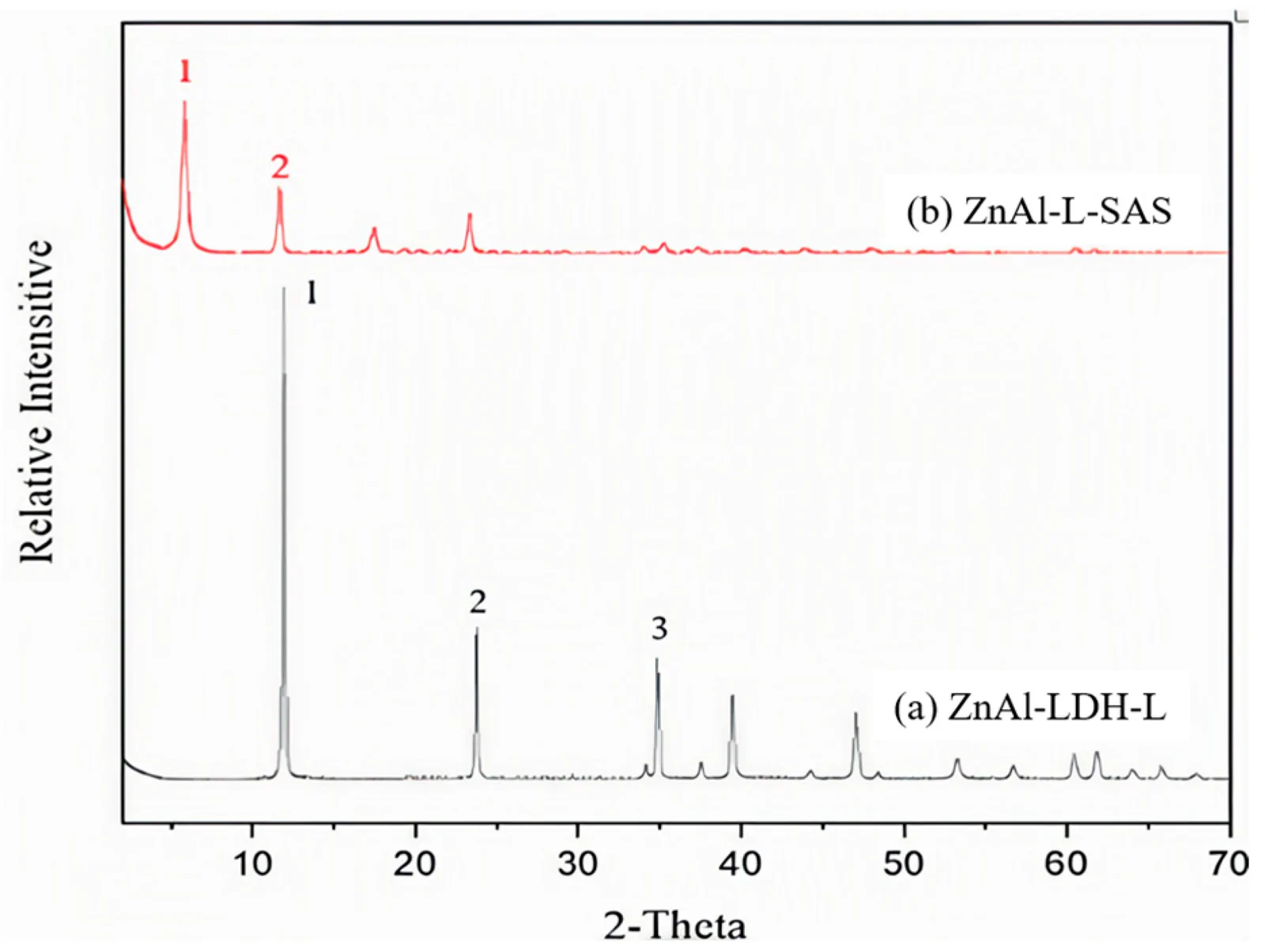
XRD patterns of (a) ZnAl-LDH-L and (b) ZnAl-LDH-L modified with SAS. Peaks 1, 2, and 3 are assigned to the (003), (006), and (009) reflections, respectively.

XRD results (Figure 5 and Figure 6; Table 2 and Table 3) demonstrate that the extent of basal-spacing expansion in LDHs is strongly governed by the molecular structure, charge distribution, and steric characteristics of the intercalating modifiers. Although all three modifiers-SAS, DMSI, and CDS—carry sulfonate groups capable of electrostatic interaction with the LDH layers, their distinct geometries lead to markedly different interlayer arrangements and thus different gallery heights.

SAS, the smallest and least sterically hindered molecule, introduces moderate expansion (d_003_ ≈ 14.5 Å). Its planar aromatic ring and -NH_2_/-SO_3_^−^ functionalities allow relatively compact packing within the gallery, forming monolayer or tilted arrangements stabilized by hydrogen bonding and ionic interactions. DMSI, containing a bulkier aromatic diester backbone, produces substantially larger spacing (d_003_ ≈ 25.0 Å). The increased gallery height reflects steric constraints that prevent tight packing, forcing the molecules into more extended or partially interdigitated configurations. CDS yields the largest expansion (d_003_ ≈ 28.1 Å), attributable to its long flexible alkyl chain and sulfonate headgroup. The chain mobility enables hydrophobic packing and partial bilayer formation, while the sulfonate group anchors the molecules electrostatically to the LDH layers, producing a highly expanded and yet ordered lamellar structure.

Therefore, MgAl–LDH intercalation is controlled by both gallery accessibility and layer charge density, whereas ZnAl–LDH intercalation is predominantly governed by intrinsic layer chemistry; consequently, increasing platelet size produces substantial benefits only in the MgAl system.

For the ZnAl–LDH (L-type) samples, the (003) reflection likewise shifts markedly from 11.88° (d = 7.45 Å) to 5.83° (d = 15.17 Å), corresponding to a basal-spacing increase of 7.72 Å. Successive (006)/(009) reflections are discernible at 11.68° (d = 7.58 Å) and 17.47° (d = 5.08 Å), respectively. The spacings satisfy d_006_ ≈ d_003_/2 and d_009_ ≈ d_003_/3, indicating preserved lamellar periodicity and ordered stacking upon intercalation, thereby confirming successful gallery expansion.

**Figure 7:**
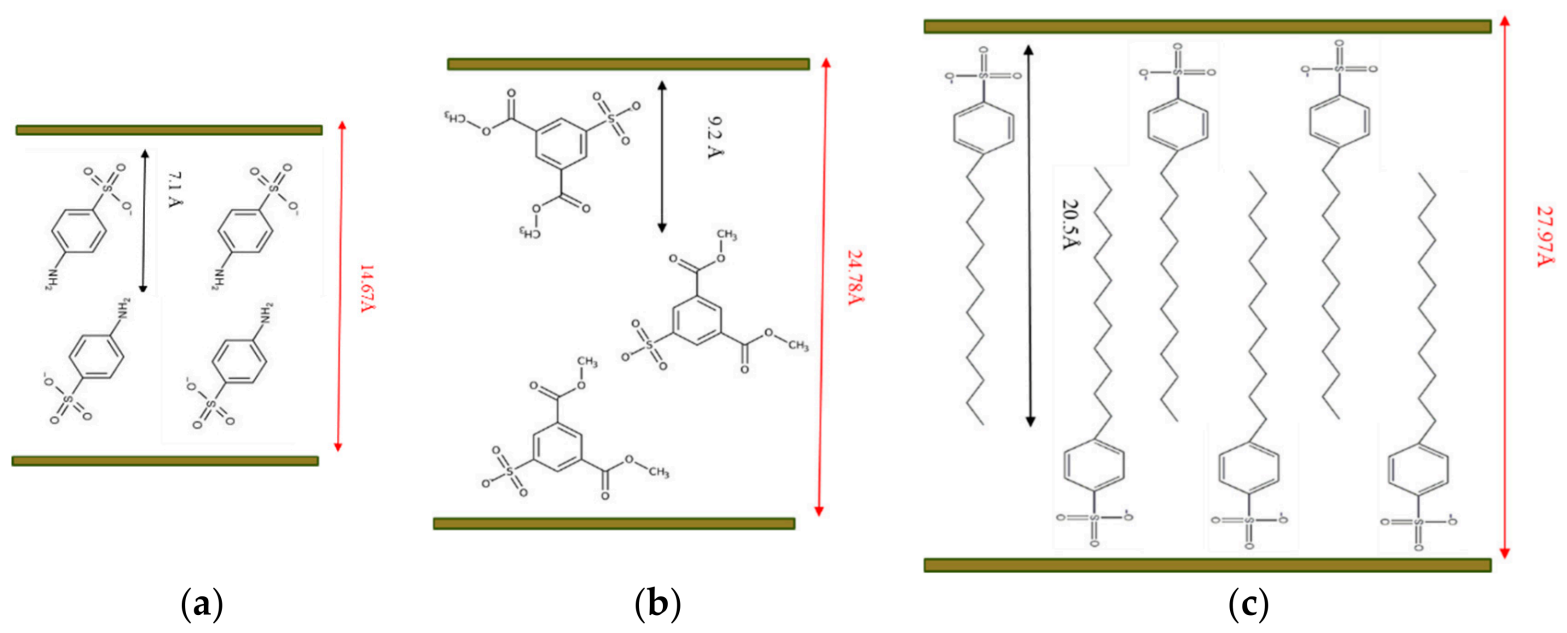
Schematic representation of (**a**) SAS, (**b**) DMSI, and (**c**) CDS intercalation modes in MgAl-LDH. These illustrations are conceptual models proposed according to XRD, FTIR, and TGA results and do not represent experimentally resolved molecular structures.

**Figure 8:**
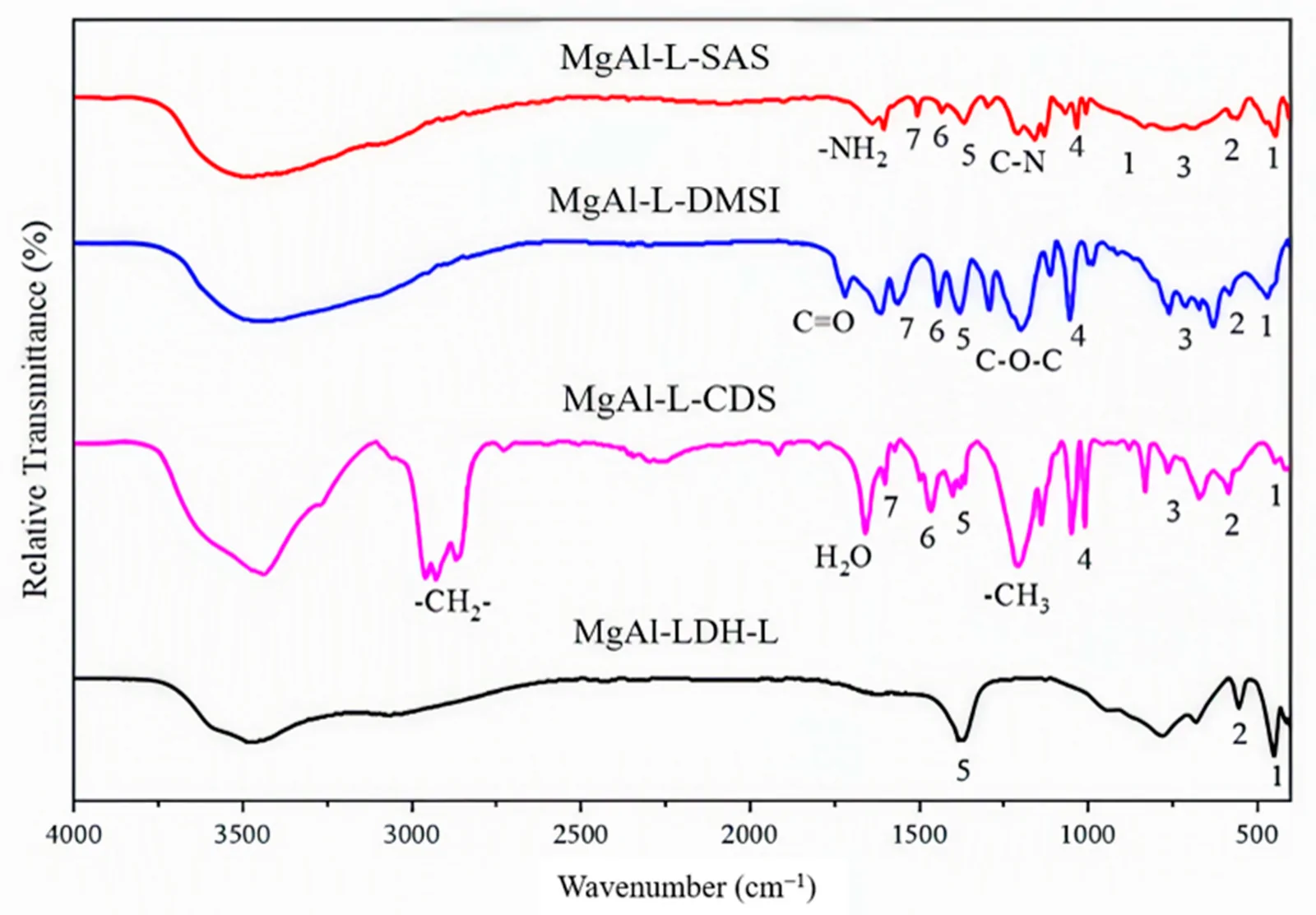
FT-IR spectra of MgAl-LDH before and after modification. The numbered peaks (1–7) indicate characteristic absorption bands assigned to different functional groups, and their corresponding assignments are summarized in Table 5.

**Figure 9:**
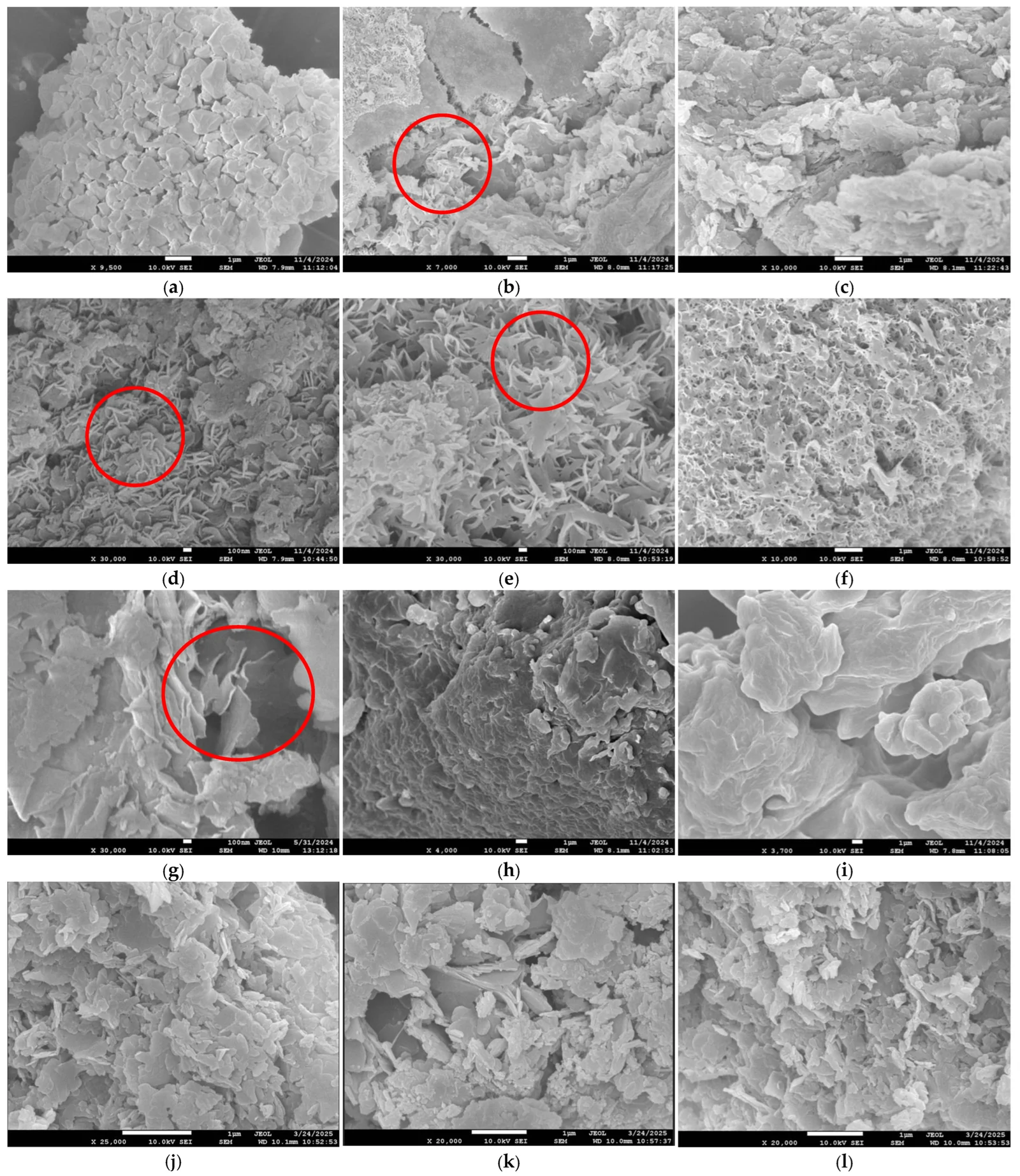
SEM images of modified LDH (**a**) MgAl-S-SAS, (**b**) MgAl-M-SAS, (**c**) MgAl-L-SAS, (**d**) MgAl-S-DMSI, (**e**) MgAl-M-DMSI, (**f**) MgAl-L-DMSI, (**g**) MgAl-S-CDS, (**h**) MgAl-M-CDS, (**i**) MgAl-L-CDS, (**j**) ZnAl-S-SAS, (**k**) ZnAl-M-SAS, (**l**) ZnAl-L-SAS. The red-circled regions in panels (**b**,**d**,**e**,**g**) highlight petal-like edge structures, which are attributed to the adsorption of intercalator molecules on the external LDH surfaces. The scale bar corresponds to 1 μm.

The intercalation modes depicted in Figure 7 are presented as schematic representations intended to illustrate plausible geometric arrangements of the modifiers within the LDH galleries. These configurations are not experimentally resolved structures but conceptual models constructed to rationalize the measured basal-spacing expansions (Table 2 and Table 3), FTIR vibrational features (Figure 8 and Figure 9), and intercalation amounts (Table 4). The schematics highlight how differences in molecular size, flexibility, and functional groups of SAS, DMSI, and CDS may lead to distinct packing motifs—ranging from compact monolayers to tilted or partially interdigitated arrangements—consistent with the structural trends observed experimentally.

FTIR spectra (Figure 8; Table 5) corroborate these structural interpretations by showing modifier-specific vibrational signatures within the LDH galleries, confirming chemical incorporation rather than surface adsorption. The presence of aromatic C=C, ester C–O–C, and long-chain -CH_2_– modes directly reflects the distinct molecular backbones and packing motifs of SAS, DMSI, and CDS.

Table 4 shows that the TGA-derived intercalation estimates collectively reveal that the degree of intercalation in LDHs is governed by a coupled dependence on platelet size and modifier molecular structure. A clear size hierarchy (L > M > S) is consistently observed across all modifiers. Large platelets possess higher crystallinity, wider and more continuous galleries, and lower defect densities, which together reduce diffusion resistance and provide more accessible interlayer pathways for incoming modifiers. In contrast, small platelets contain more edge defects and fragmented galleries, increasing the likelihood of surface adsorption rather than true intercalation and thereby limiting the achievable loading.

Modifier chemistry further introduces distinct geometric and steric constraints that determine how each molecule packs within the LDH galleries. CDS, bearing a flexible long alkyl chain and a sulfonate headgroup, achieves the highest intercalation degree. Its chain mobility allows hydrophobic packing and partial bilayer formation, while the sulfonate group anchors the molecule electrostatically to the LDH layers, producing both high loading and substantial basal-spacing expansion. SAS, with a smaller aromatic ring and -NH_2_/-SO_3_^−^ functionalities, intercalates in more compact monolayer or tilted configurations stabilized by ionic and hydrogen-bonding interactions, resulting in moderate gallery expansion and intermediate loading. DMSI, containing a rigid aromatic diester backbone, experiences significant steric hindrance during insertion; its bulky geometry restricts packing efficiency and forces more extended or sparsely populated arrangements, yielding the lowest intercalation degree despite its strong sulfonate functionality.

The combined trends (L > M > S and CDS > SAS > DMSI) highlight the interplay between gallery accessibility and modifier geometry. Large platelets provide structurally coherent galleries capable of accommodating modifiers with greater molecular flexibility, while small platelets impose steric and diffusion limitations that disproportionately affect bulky or rigid modifiers such as DMSI. Consequently, pairing large platelets with flexible, highly polar modifiers—exemplified by CDS—maximizes intercalation efficiency and produces the most expanded and ordered lamellar structures. These structural advantages establish a robust platform for improved dispersion and enhanced thermal, mechanical, and optical performance in the resulting PMMA nanocomposites.

Comparison of the SAS intercalation amounts across the six LDH samples (MgAl-LDH S/M/L SAS and ZnAl-LDH S/M/L SAS) reveals a pronounced contrast between the two LDH systems. In the MgAl-LDH series, the intercalation degree increases significantly with platelet size (L > M > S), whereas in the ZnAl-LDH series the SAS loading remains largely insensitive to lateral size, with S, M, and L platelets exhibiting comparable intercalation levels.

For MgAl-LDH, the strong size dependence arises from the cooperative influence of crystallinity, defect density, and gallery continuity. Small MgAl platelets contain a higher fraction of edge defects, broken layers, and aggregated domains, which promote surface adsorption and restrict deep gallery penetration of SAS, resulting in lower intercalation degrees. As the platelet size increases, the LDH layers become more continuous and better ordered, with fewer structural defects and more extended galleries. These structural improvements reduce diffusion resistance and allow the higher layer charge density of MgAl–LDH to be more fully compensated by SAS, producing substantially higher intercalation in the L-type samples. Thus, MgAl-LDH operates in a regime where both layer chemistry and platelet size jointly govern intercalation efficiency.

In contrast, ZnAl-LDH exhibits a much lower layer charge density and weaker layer–anion affinity than MgAl-LDH. Under these conditions, the driving force for SAS intercalation is dominated by intrinsic layer chemistry rather than geometric accessibility. Increasing the lateral size from S to M to L does not significantly alter the number of anionic sites requiring compensation or the effective interaction strength between SAS and the Zn–O–Al layers. Moreover, the higher interlayer water content and lower polarity of ZnAl-LDH limit the achievable packing density of SAS, even when gallery continuity improves with increasing platelet size. As a result, SAS loading in ZnAl-LDH is only weakly modulated by platelet size and remains comparatively constant across S, M, and L samples.

Taken together, these results demonstrate that MgAl-LDH is in a size-sensitive intercalation regime, where larger platelets provide structurally superior galleries that enable higher anion uptake consistent with its higher layer charge density. In contrast, ZnAl-LDH is in a size-insensitive intercalation regime, where the inherently lower layer charge and weaker layer–anion interactions dominate the intercalation behavior, rendering geometric factors such as lateral size secondary. This fundamental distinction explains why SAS intercalation in MgAl-LDH exhibits a pronounced size hierarchy (L > M > S), whereas ZnAl-LDH shows nearly size-independent intercalation across all platelet sizes. Overall, the combined XRD, FTIR, and TGA evidence establishes a coherent structure–property relationship: interlayer spacing and intercalation amount are jointly dictated by modifier geometry and platelet crystallinity, which together determine the achievable packing configuration within the LDH galleries. These structural factors ultimately govern dispersion behavior and the multi-property enhancements observed in the PMMA nanocomposites.

The morphologies of the modified LDHs were examined using SEM for nine representative samples, as shown in Figure 9. The images reveal that the surface features of the LDHs vary substantially depending on both the modifier chemistry and the platelet size. For MgAl-LDH, the SAS-modified samples (Figure 9a–c) show only slight petal-like edges in the medium- and large-platelet specimens, as shown in the red cycle, consistent with their relatively low intercalation amounts and the tendency of SAS to reside predominantly within the galleries rather than on the external surfaces. In contrast, the DMSI-modified MgAl-LDHs (Figure 9d–f) exhibit extensive petal-like edge structures across all platelet sizes. This morphology indicates that DMSI molecules preferentially adsorb on the platelet edges rather than intercalate into the galleries, in agreement with their low intercalation degrees. The CDS-modified MgAl-LDHs (Figure 9g–i) display the opposite trend: only the small-platelet sample shows minor petal-like features, whereas the medium- and large-platelet samples exhibit smooth surfaces without edge decoration. This observation correlates with the significantly higher intercalation amounts of CDS, suggesting that most CDS molecules reside within the LDH galleries rather than on the external surfaces.

A similar analysis of the ZnAl-LDH samples modified with SAS (Figure 9 j–l) shows petal-like features across all platelet sizes, and the platelet stacking appears more open and less compact than in the MgAl-LDH counterparts. These differences highlight the strong influence of modifier chemistry and LDH layer composition on the resulting morphology.

Distinct morphological behaviors can be rationalized by considering the molecular structures and interaction characteristics of the three modifiers. SAS contains a para-substituted benzene ring with sulfonate and amine groups, giving moderate polarity, small molecular size, and reasonable hydrogen-bonding capability. These features allow SAS to intercalate into LDH galleries with moderate efficiency, generally higher than DMSI but lower than CDS. DMSI possesses two ester groups and a rigid aromatic backbone, resulting in a bulky and sterically hindered structure that limits its ability to enter the galleries; consequently, DMSI primarily adsorbs on platelet edges and exhibits the lowest intercalation amounts. CDS, despite containing an aromatic moiety, has a more flexible molecular structure and higher polarity, enabling strong ionic interactions with the LDH layers. These characteristics promote efficient intercalation and yield the highest loading among the three modifiers.

Overall, these findings demonstrate that intercalation efficiency in modified LDHs is jointly controlled by platelet size and modifier molecular structure, with the relative importance of each factor determined by the intrinsic layer chemistry. MgAl-LDH exhibits a cooperative size–chemistry relationship, whereas ZnAl-LDH is dominated by layer-chemical constraints. Modifier polarity, functional-group complementarity, molecular volume, and flexibility are key determinants of intercalation behavior, with highly polar, flexible, and compact molecules such as CDS achieving the most effective intercalation across all LDH systems.

### 2.3. PMMA/LDH Nanocomposites

#### 2.3.1. Dispersion Morphology of LDH in PMMA Matrix

**Figure 10:**
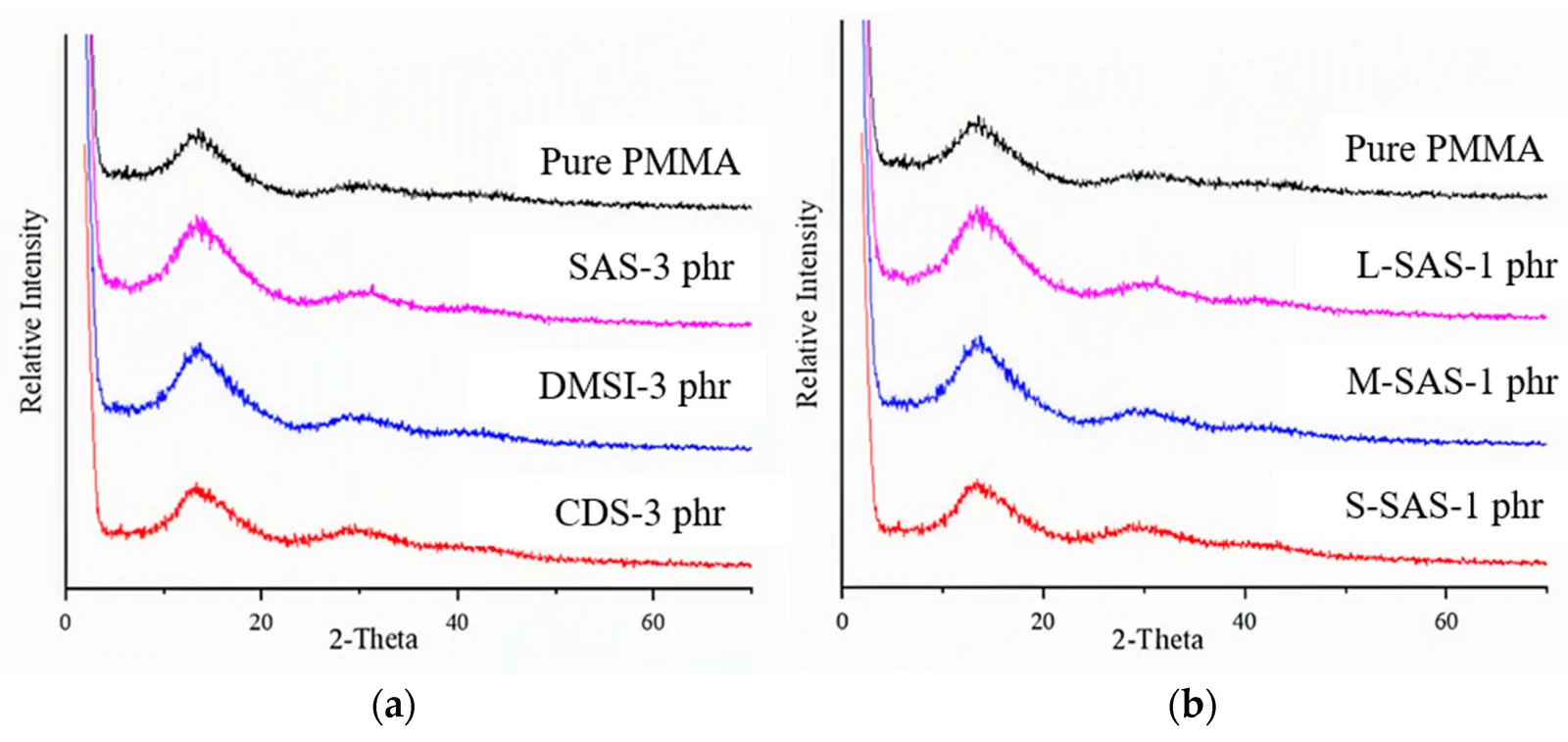
XRD patterns of pristine PMMA and PMMA/modified LDH: (**a**) MgAl-LDH-L, (**b**) ZnAl-LDH.

To evaluate the dispersion state of modified LDHs within the PMMA matrix, PMMA/LDH nanocomposites were prepared via emulsion polymerization using MMA monomer and three types of organically modified LDHs. Figure 10 presents the XRD patterns of MgAl-LDH nanocomposites containing 3 phr of SAS-, DMSI-, or CDS-modified LDHs with large platelet size. In the low-angle region (2θ ≈ 3–5°), no discernible LDH lamellar reflections such as (003), (006), or (009) are detected. The absence of long-range gallery stacking indicates that the LDH platelets are highly dispersed within the PMMA matrix, forming thin and short stacks rather than extended lamellar aggregates. Although XRD confirms the loss of ordered LDH stacking, it cannot unambiguously distinguish whether the resulting nanocomposites are fully exfoliated or partially intercalated.

**Figure 11:**
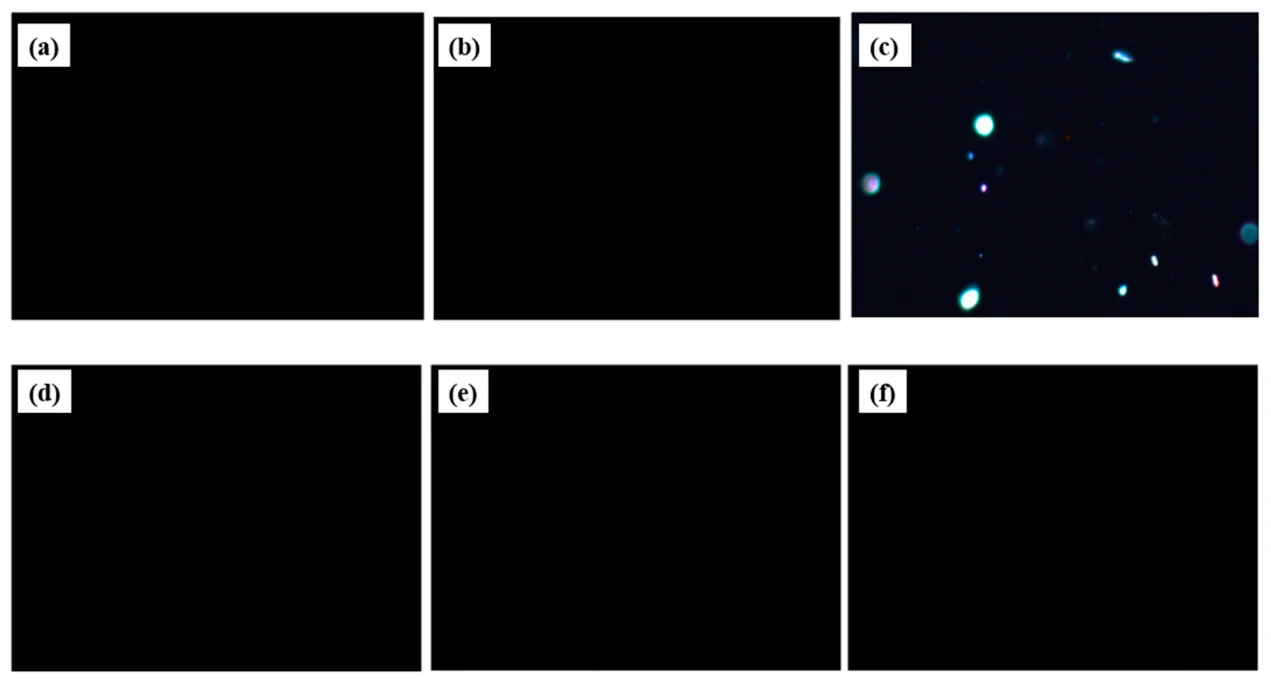
POM images of PMMA/modified MgAl-LDH-L-3 phr (**a**) SAS (**b**) DMSI (**c**) CDS and PMMA/modified ZnAl-LDH-SAS-1 phr (**d**) Large (**e**) Medium (**f**) Small. Bright spots correspond to birefringent LDH aggregates arising from local phase separation and reduced compatibility between CDS-modified LDH and PMMA.

To further assess the uniformity of LDH dispersion, polarized optical microscopy (POM) was employed. As shown in Figure 11a–c, PMMA/MgAl-LDH nanocomposites modified with SAS and DMSI exhibit no birefringent crystalline spots, suggesting homogeneous dispersion of LDH platelets throughout the polymer matrix. In contrast, the CDS-modified MgAl-LDH composite displays distinct bright crystalline domains, indicating localized phase separation. A similar trend is observed for PMMA/ZnAl-LDH nanocomposites (Figure 11d–f), where SAS-modified ZnAl-LDH samples show no crystalline spots, confirming uniform dispersion. These results demonstrate that CDS-modified LDH, despite its high intercalation amount, possesses long alkyl chains that render the LDH surface more hydrophobic, thereby reducing compatibility with PMMA and promoting phase separation. In comparison, SAS- and DMSI-modified LDHs maintain better interfacial affinity with PMMA, resulting in more uniform dispersion.

**Figure 12:**
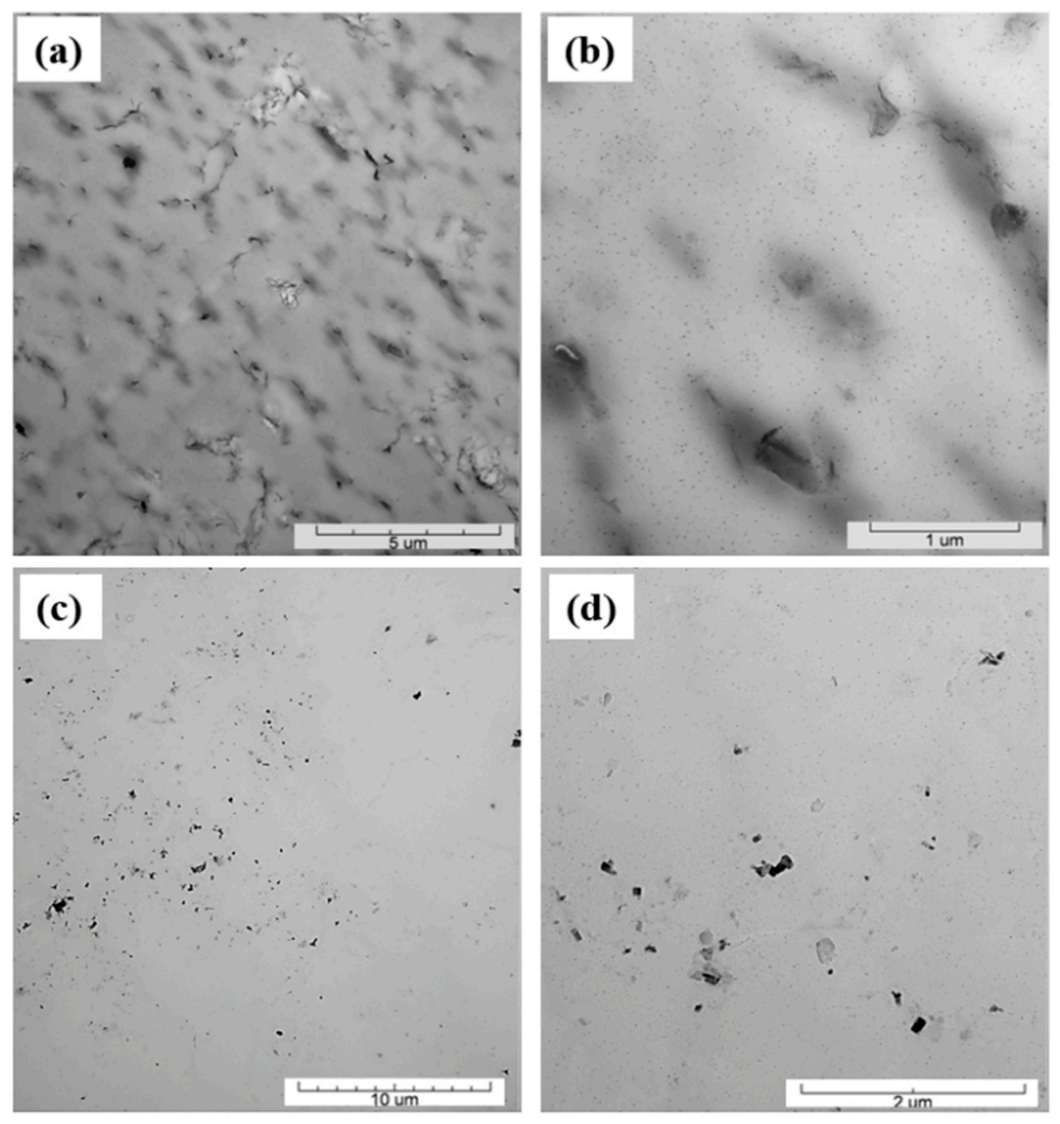
TEM images of PMMA/MgAl–M-SAS (3 phr) nanocomposites at magnifications of (**a**) 5 k and (**b**) 20 k, and PMMA/MgAl-L-CDS (3 phr) nanocomposites at magnifications of (**c**) 2.5 k and (**d**) 15 k.

Transmission electron microscopy (TEM) provides the most direct evidence of LDH dispersion within the PMMA matrix. Figure 12a,b shows the TEM micrographs of the PMMA/MgAl–LDH-M-SAS (3 phr) nanocomposite, in which the LDH platelets are observed as thin, well-distributed nanosheets without the presence of large aggregates. This morphology confirms that SAS modification facilitates effective exfoliation and promotes uniform dispersion of LDH platelets throughout the PMMA matrix.

In contrast, the TEM images of the PMMA/MgAl–LDH-L-CDS (3 phr) nanocomposite reveal noticeably aggregated LDH domains embedded within the polymer matrix, as shown in Figure 12c,d. These larger, clustered features are consistent with the birefringent crystalline spots observed in POM, indicating localized phase separation. The aggregation behavior is attributed to the long alkyl chain of CDS, which increases the hydrophobicity of the LDH surface and reduces compatibility with PMMA, thereby limiting the extent of nanoscale dispersion despite the high intercalation degree achieved in the LDH galleries.

Collectively, the XRD, POM, and TEM analyses reveal that the dispersion behavior of LDH platelets in PMMA is strongly influenced by the molecular structure of the modifier. SAS and DMSI yield uniform dispersion due to their moderate polarity and favorable interfacial interactions with PMMA, whereas CDS, with its long hydrophobic alkyl chain, tends to induce phase separation despite achieving the highest intercalation degree in LDH galleries. These findings highlight the critical role of modifier chemistry in governing the compatibility between LDH platelets and the PMMA matrix, ultimately determining the dispersion quality and nanocomposite morphology.

#### 2.3.2. Structure–Property Relationships of PMMA/LDHs Nanocomposites

**Figure 13:**
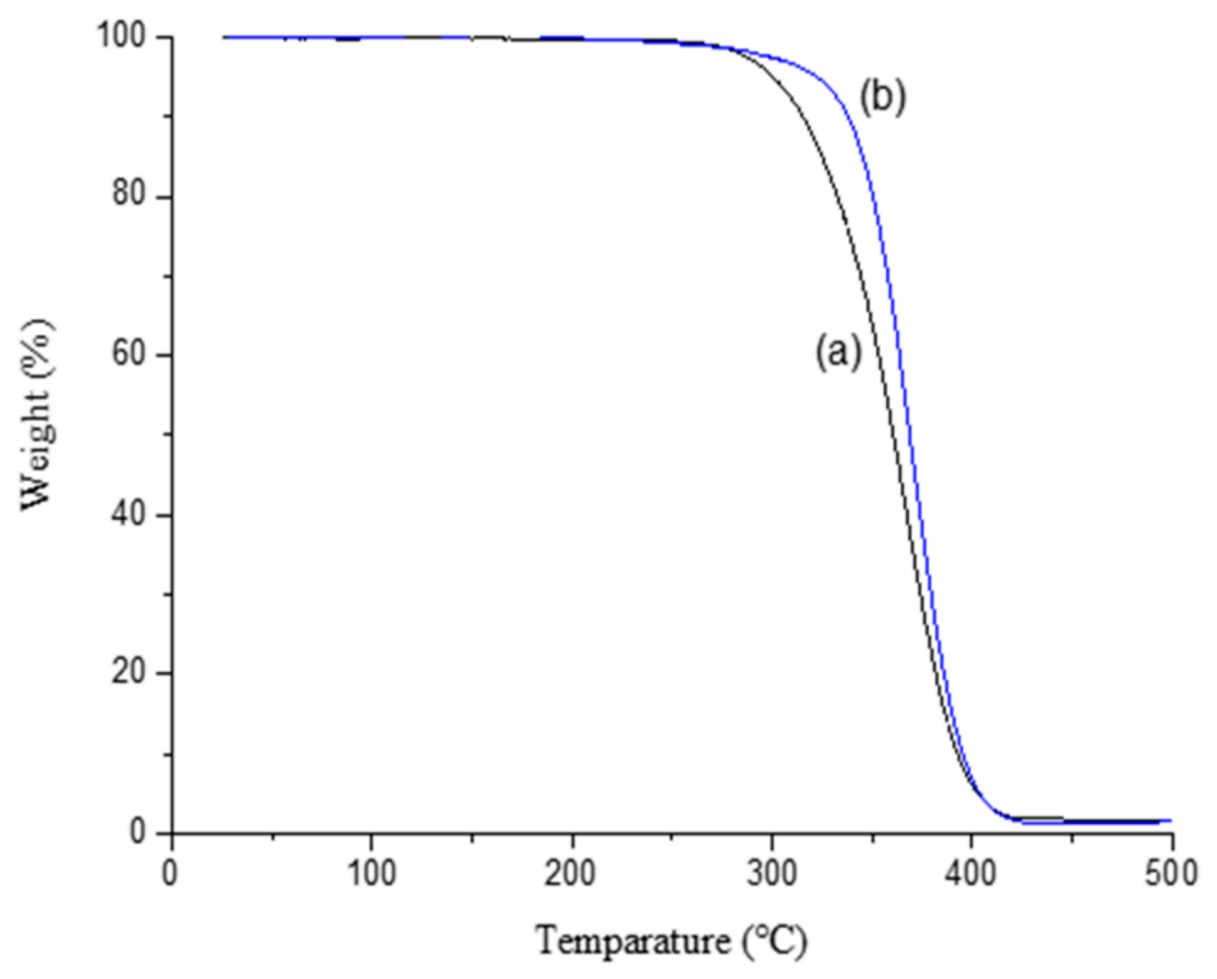
TGA curves: (a) pure PMMA and (b) PMMA/MgAl-M-SAS-3 phr nanocomposite obtained under an air atmosphere.

**Figure 14:**
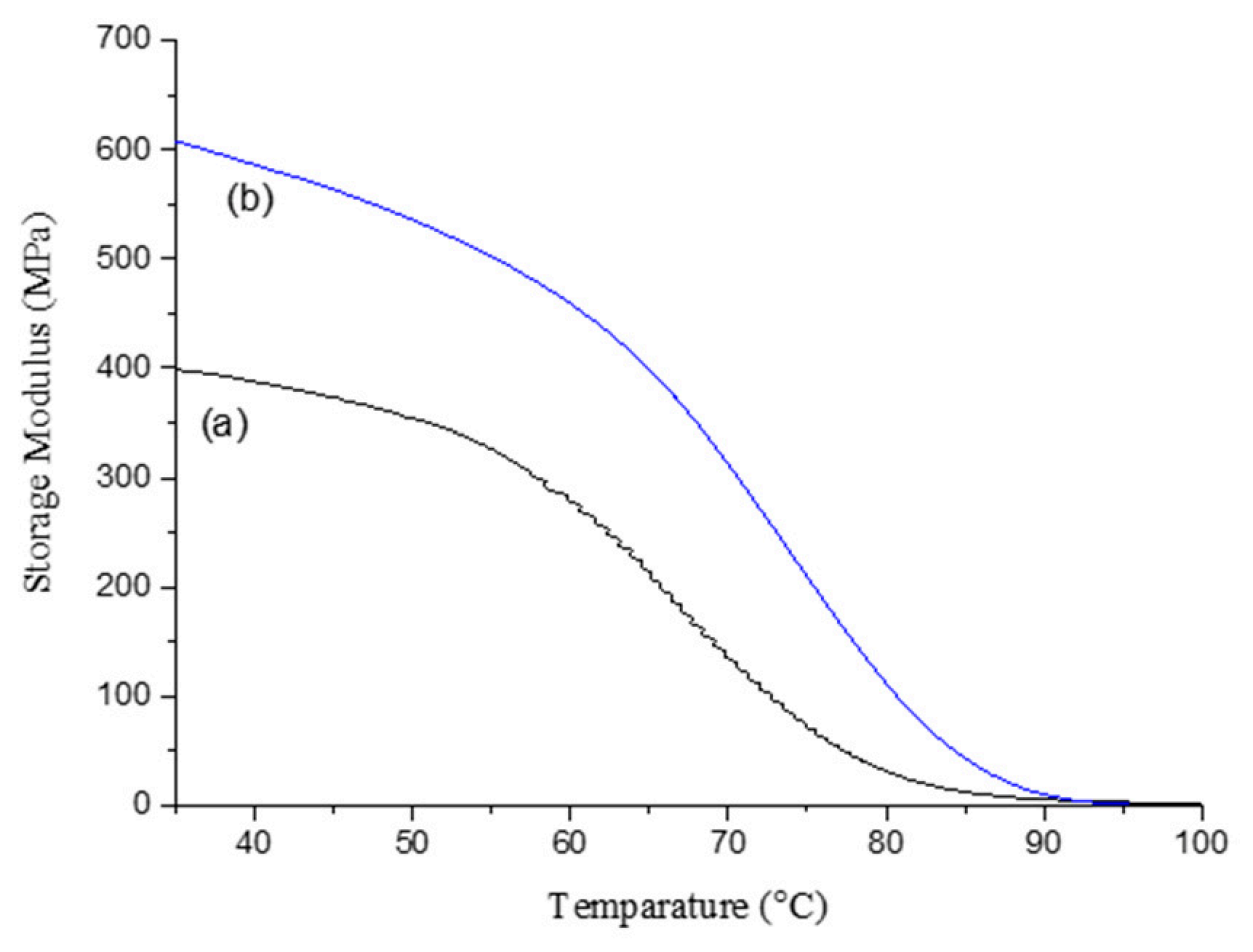
Storage modulus (E′) of (a) pure PMMA and (b) PMMA/MgAl-M-SAS-3 phr nanocomposite.

**Figure 15:**
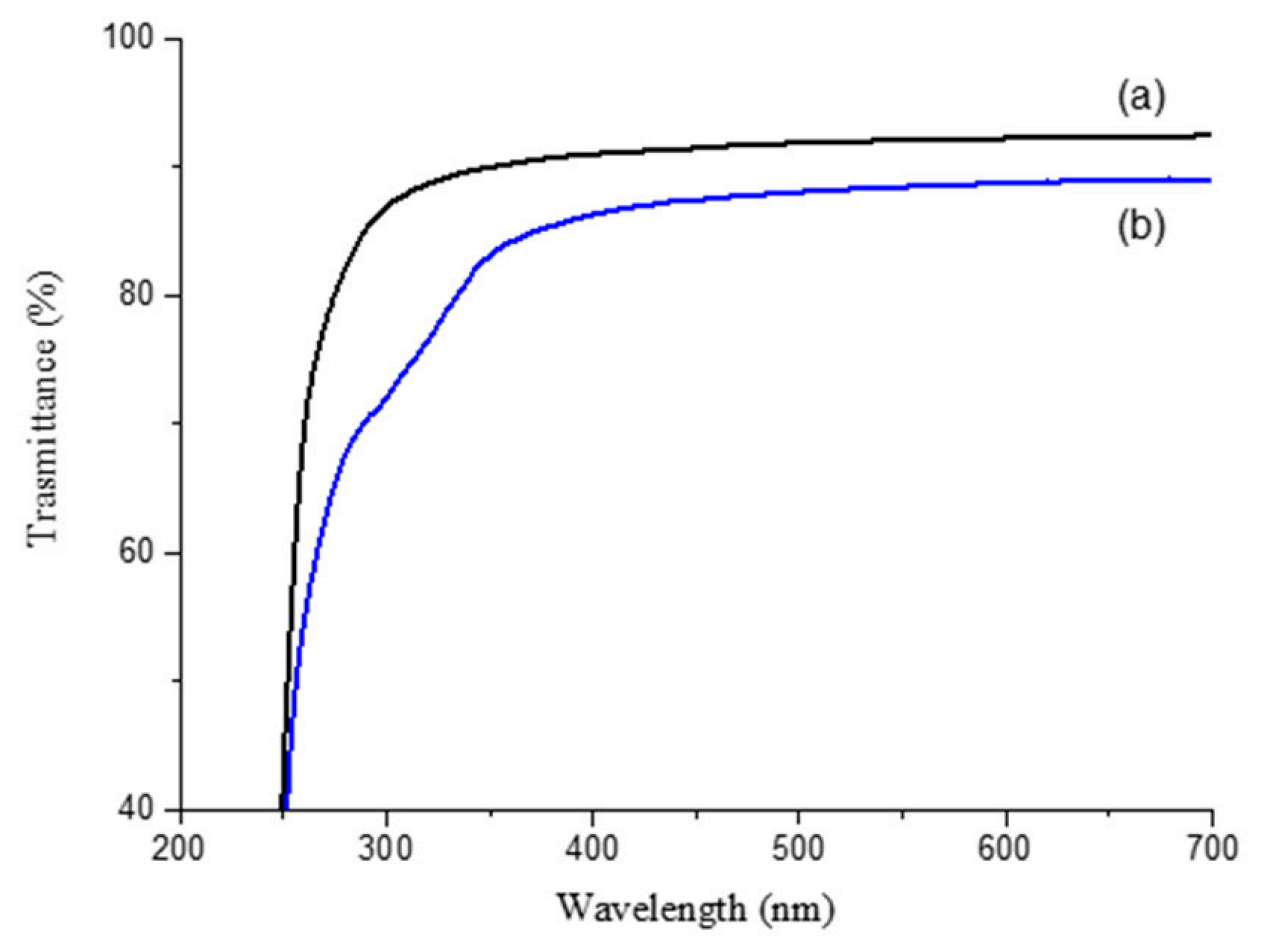
UV–Visible optical transmittance spectra of (a) pure PMMA and (b) the PMMA/MgAl-M-SAS nanocomposite containing 3 phr of MgAl-M-SAS.

To further investigate the thermal, mechanical, and optical properties of PMMA/LDH nanocomposites, four representative LDH modifiers were selected based on previously reported dispersion behavior and intercalation characteristics [18,20]. Three of these, MgAl-M-SAS, ZnAl-S-SAS, and ZnAl-M-SAS, exhibit favorable dispersion within the PMMA matrix and moderate intercalation levels (approximately 60%). In contrast, MgAl-L-CDS was chosen as a comparative system due to its markedly poorer dispersion yet exceptionally high intercalation amount (up to 97%). To facilitate interpretation and improve data transparency, representative TGA, DMA, and UV–Visible spectra of neat PMMA and selected PMMA/LDH nanocomposites are provided in Figure 13, Figure 14 and Figure 15. These characterization results demonstrate how the thermal stability (T_5d_), storage modulus, and optical transmittance values reported in Table 6 were obtained from the original experimental measurements. The complete thermal, mechanical, and optical performance dataset is summarized in Table 6, allowing a systematic evaluation of the relationships between LDH platelet dispersion, intercalation characteristics, filler loading, and the resulting nanocomposite properties.

Using these four LDH compositions, PMMA nanocomposites were prepared via emulsion polymerization at various LDH loadings. This design enables a systematic evaluation of how LDH dispersion state, intercalation amount, and platelet size collectively influence the resulting composite properties. The selected systems provide a balanced framework for correlating structural parameters—such as gallery expansion, platelet delamination, and interfacial compatibility—with macroscopic performance indicators, including thermal stability, mechanical reinforcement, and optical transparency.

**Figure 16:**
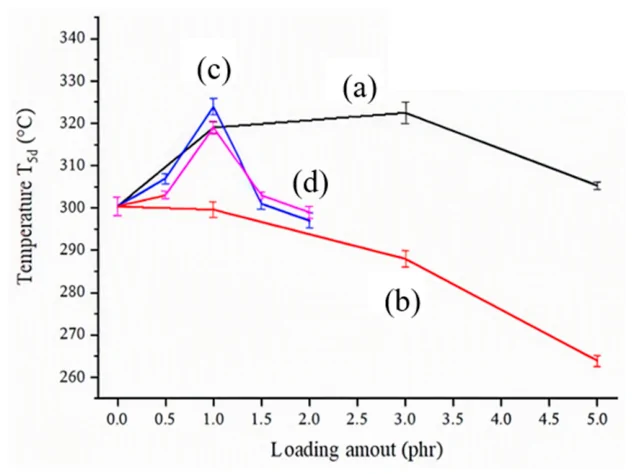
T_5d_ (temperature at 5% weight loss) of PMMA/LDH nanocomposites as a function of LDH loading for four modified LDH systems: (a) MgAl-M-SAS, (b) MgAl-L-CDS, (c) ZnAl-M-SAS, and (d) ZnAl-S-SAS.

Across all modifiers, incorporation of 3 phr MgAl-LDH leads to a measurable enhancement in the thermal stability of PMMA. Among the tested formulations, PMMA/MgAl-M-SAS-3 phr exhibits the most pronounced improvement, with its T_5d_, defined as the temperature at which a material has lost 5% of its initial mass during thermogravimetric analysis (TGA), increasing to 322.5 °C, compared with 300.4 °C for neat PMMA, as shown in Figure 13 and Figure 16 and Table 6. This shift reflects simultaneous elevation of both the onset and subsequent degradation thresholds, indicating that the medium-sized SAS-modified platelets provide the most effective barrier against thermal decomposition.

Among the evaluated MgAl-LDH systems, PMMA/MgAl-M-SAS-3 phr exhibited superior thermal stability compared with PMMA/MgAl-L-CDS-3 phr. This result suggests that nanoscale dispersion and interfacial compatibility may exert a greater influence on thermal performance than intercalation degree alone. This composition is therefore selected as a representative system for correlating mechanical reinforcement and optical performance, enabling clearer interpretation of how platelet size and interfacial chemistry govern the final composite properties.

For the ZnAl system, the most effective formulation is the SAS-modified medium-sized platelet composite at 1 phr, which achieves a T_5d_ of 324 °C, substantially higher than neat PMMA (300.4 °C). At this low loading, the medium platelet size again provides the most favorable balance between interlayer expansion and interfacial coverage. This promotes uniform dispersion, suppresses local aggregation, and enhances thermal stability while maintaining desirable optical and mechanical baselines.

The improved performance at 1 phr also reflects the inherently lower layer charge density of ZnAl-LDH, which reduces the need for higher filler content to achieve effective interfacial interactions. Consequently, the ZnAl-M-SAS/1 phr formulation represents the optimal condition for maximizing thermal stability in the ZnAl-based nanocomposites [21,22].

In contrast, the PMMA/MgAl-L-CDS nanocomposite exhibits inferior thermal stability relative to neat PMMA. The long alkyl chain of the CDS modifier renders the LDH surface highly hydrophobic, significantly reducing its compatibility with the PMMA matrix. As a result, the LDH platelets disperse poorly and form aggregated domains, which shorten the effective heat-transfer pathway compared with well-dispersed systems. Upon heating, these aggregates facilitate more rapid thermal transport through the composite. In addition, the long alkyl chains of CDS possess inherently lower thermal resistance. Poor nanoscale dispersion and LDH agglomeration are considered the dominant factors responsible for the reduced thermal stability, whereas the lower thermal resistance of the CDS alkyl chains plays a secondary role.

The following section examines how the mechanical properties of PMMA/LDH nanocomposites are influenced by the LDH dispersion state, intercalation amount, and platelet size. By correlating these structural parameters with the resulting macroscopic performance, we aim to elucidate the structure–property relationships that govern mechanical reinforcement in PMMA-based LDH nanocomposites.

**Figure 17:**
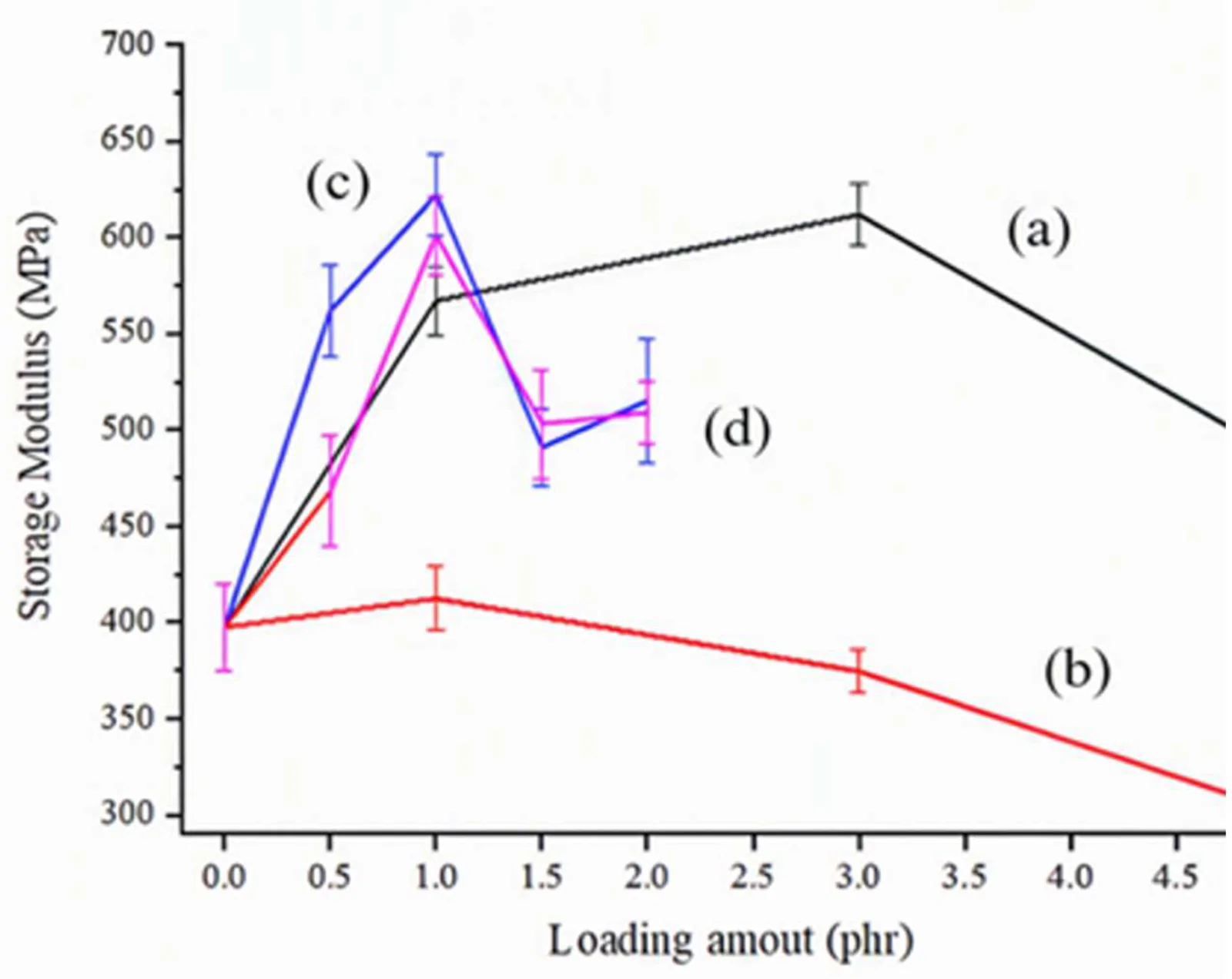
Storage modules of PMMA nanocomposites containing different modified LDHs as a function of LDH loading: (a) MgAl–M–SAS, (b) MgAl–L–CDS, (c) ZnAl–M–SAS, and (d) ZnAl–S–SAS.

In Figure 14 and Figure 17, the dynamic mechanical analysis reveals that the storage modulus (E′) of all LDH-modified PMMA systems exceeds that of neat PMMA (397.4 ± 23 MPa). The most significant enhancements are observed for PMMA/MgAl-M-SAS-3 phr and PMMA/ZnAl-SAS-M-1 phr, which reach 611.7 ± 16 MPa and 622 ± 21 MPa, respectively—representing increases of approximately 54–56% relative to pure PMMA. These improvements reflect the favorable dispersion and moderate intercalation levels achieved in these systems, which promote efficient stress transfer between the polymer matrix and LDH platelets. In contrast, PMMA/MgAl-L-CDS-3 phr exhibits a markedly reduced modulus (375.6 ± 11 MPa), consistent with its poor dispersion and aggregated morphology. The ZnAl-S-SAS-0.5 phr sample shows an intermediate modulus of 468 ± 29 MPa, indicating partial reinforcement under limited loading and shorter modification time. Collectively, these results underscore the critical roles of platelet size, modifier chemistry, and reaction conditions in determining the extent of mechanical reinforcement achievable in PMMA/LDH nanocomposites, as shown in Table 6.

**Figure 18:**
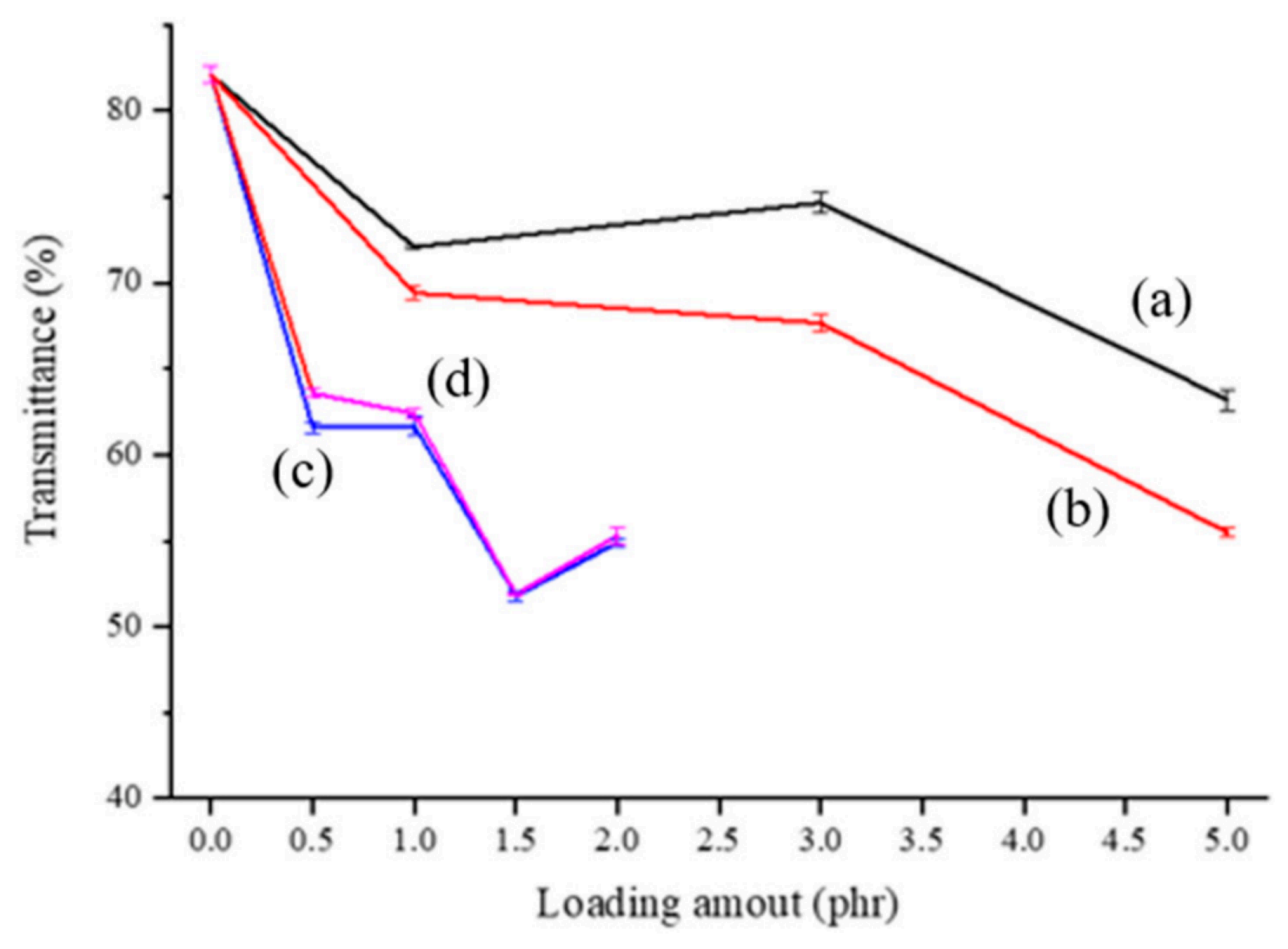
Optical transmittance at a wavelength of 280 nm of PMMA nanocomposites incorporating different modified LDHs at various loading levels: (a) MgAl-M-SAS, (b) MgAl-L-CDS, (c) ZnAl-M-SAS, and (d) ZnAl-S-SAS.

The optical transmittance behavior of PMMA/LDH nanocomposites reveals clear structure-property relationships governed by LDH platelet size, intercalation amount, and dispersion quality. As shown in Figure 15 and Figure 18, LDH incorporation significantly reduces UV transmittance while largely preserving visible transparency. Among the MgAl-based systems, PMMA/MgAl-M-SAS exhibited the highest visible-light transmittance (88.8%) together with relatively moderate attenuation in the UVA (81.6%) and UVB (74.7%) regions. In contrast, ZnAl-based systems displayed stronger UV blocking, with PMMA/ZnAl-M-SAS showing lower transmittance values of 68.8% and 61.7% in the UVA and UVB regions, respectively.

These trends can be attributed primarily to differences in platelet size and intercalation amount. The larger platelet dimensions of ZnAl-LDHs (200–400 nm) enhance UV attenuation through Mie scattering, as their sizes approach UV wavelengths, resulting in higher scattering efficiency and lower UV transmittance [23]. In addition, LDHs containing higher amounts of intercalated aromatic modifiers provide further UV shielding through π → π* and n → π* electronic transitions of the benzene rings, contributing to absorption in both the UVA and UVB regions [24]. Consequently, UV-blocking performance is enhanced through the combined effects of scattering and absorption.

At a loading of 3 phr, all PMMA/LDH nanocomposites maintained high transparency at 550 nm while substantially reducing UV transmission. PMMA/MgAl-M-SAS-3 phr showed only a slight decrease in visible transmittance (92.1% to 88.8%) but exhibited stronger attenuation in the UVA (88.7% to 81.6%) and UVB (82.1% to 74.7%) regions. The strongest UV shielding among the MgAl systems was observed for PMMA/MgAl-L-CDS-3 phr, with reductions of 3.6% at 550 nm, 12.1% at 320 nm, and 14.4% at 280 nm. Overall, the MgAl series showed reductions of 3.3–3.6% at 550 nm, 7.1–12.1% at 320 nm, and 7.4–14.1% at 280 nm, indicating effective UV protection without significant loss of transparency.

A similar loading-dependent trade-off was observed for the ZnAl-SAS series. At 1.5 phr, transmittance decreased to 81.8 ± 0.5% at 550 nm, accompanied by strong UV attenuation at 320 nm (62.3 ± 0.6%) and 280 nm (50.4 ± 0.4%). Reducing the loading to 0.5 phr improved visible transmittance to 88.3 ± 0.3% while maintaining substantial UV blocking (73.1 ± 0.1% at 320 nm and 63.6 ± 0.2% at 280 nm).

Overall, PMMA/MgAl-M-SAS-3 phr provided the best balance between visible transparency and UV shielding, whereas PMMA/MgAl-L-CDS-3 phr and ZnAl-SAS-1.5 phr delivered the strongest UV-blocking performance. These results demonstrate that UV-shielding behavior can be effectively tailored through the combined control of LDH platelet size, intercalation amount, and dispersion state.

## 3. Materials and Methods

Methyl methacrylate (MMA) monomer was supplied by Kaohsiung Monomer Corporation, Kaohsiung, Taiwan. Magnesium Nitrate [Mg(NO_3_)_2_], Zinc Nitrate [Zn(NO_3_)_2_] and Aluminum Nitrate [Al(NO_3_)_3_] were purchased from SHOWA, Tokyo, Japan. Sulfanilic acid sodium salt hydrate (SAS) was obtained from Sigma-Aldrich, St. Louis, MO, USA. Sodium dimethyl 5-sulfoisophthalate (DMSI) and calcium dodecylbenzene sulfonate (CDS) were purchased from Tokyo Chemical Industry (TCI), Tokyo, Japan. The emulsifier, disodium cocoamphodipropionate, K2, was obtained from Arxada AG, Basel, Switzerland. Potassium persulfate, KPS, was purchased from J. T. Baker, Phillipsburg, NJ, USA.

### 3.1. Preparation of MgAl–LDH/ZnAl–LDH (L/M/S)

MgAl–LDH/ZnAl–LDH were synthesized via the co-precipitation method. A mixed nitrate solution was prepared with a molar ratio of Mg(NO_3_)_2_/Zn(NO_3_)_2_:Al(NO_3_)_3_ = 2:1, dissolved in deionized water. Separately, an alkaline solution of 2.0 M NaOH and 0.5 M NaHCO_3_ was prepared. Under vigorous stirring, the two solutions were slowly combined to maintain the reaction medium at pH 10, allowing uniform suspension of the precursors. The mixture was then transferred into a Teflon-lined autoclave and hydrothermally treated at 100 °C for 6, 15, and 24 h, yielding LDH products with average platelet sizes of approximately 50, 100, and 200 nm, respectively. Longer hydrothermal treatment promotes Ostwald ripening and crystal growth, resulting in larger and more crystalline LDH platelets [25,26].

### 3.2. Preparation of Surface-Modified MgAl/ZnAl–LDH (L/M/S)

Surface modification was carried out using the reconstruction method. As-prepared LDH was first calcined to obtain MgAl/ZnAl layered double oxide (LDO). A threefold anion-exchange capacity (AEC) of LDH modifiers—including sulfanilic acid sodium salt hydrate (SAS), sodium dimethyl 5-sulfoisophthalate (DMSI), or calcium dodecylbenzene sulfonate (CDS)—was dissolved in 50 mL of deionized water, followed by the slow addition of 2 g of LDO under continuous stirring. The nucleation time was adjusted to 1, 5, and 10 min to control intercalation and reconstruction. The suspension was subsequently transferred into a PTFE-lined autoclave for hydrothermal treatment, allowing the LDO to reconstruct into organo-intercalated LDH via its “memory effect.” The product was washed with deionized water and centrifuged three times to remove residual species, then freeze-dried and ground into a fine white powder using an agate mortar [27].

### 3.3. Preparation of PMMA/LDH Nanocomposites

PMMA/LDH nanocomposites were synthesized via emulsion polymerization. The organo-modified LDH (3 phr) was dispersed in deionized water and stirred for 1 h, followed by the addition of 1 g of emulsifier K2 and inorganic filler, with continued stirring at 200 rpm for another hour. An initial 1 g portion of MMA was introduced as a seed emulsion precursor to facilitate LDH surface wetting and promote stable emulsion formation, and the reaction flask was placed in an oil bath at 60–65 °C under nitrogen purge for 1 h. Potassium persulfate (KPS), dissolved in 20 mL deionized water, was then added as the initiator. The remaining MMA was subsequently fed into the reactor to complete the polymerization process for about 3 h. After polymerization, the flask was cooled to room temperature while stirring, and the product was precipitated into 100 mL of methanol and stirred for 24 h to remove unreacted species. The precipitate was collected by ultracentrifugation, Sorvall C5CPLUS (Thermo Scientific, Waltham, MA, USA), (8000 rpm, 15 min), redispersed in deionized water, stirred for 15 min, and subjected to three centrifugation–washing cycles. Finally, the obtained precipitate was freeze-dried and ground into powder to yield PMMA/LDH nanocomposites.

### 3.4. Characterization

X-ray diffraction (XRD) patterns were obtained using a Bruker D8 Advance ECO (Bruker, AXS GmbH, Karlsruhe, Germany) using Cu Kα radiation (λ = 1.5406 Å, 40 kV, 25 mA) over a 2θ range of 2–80° with a step size of 0.02°, a counting time of 1.2°/sec, and a slit size of 1/4. Fourier transform infrared spectroscopy (FTIR, JASCO-4200 Type A, JASCO Inc., Tokyo, Japan) was employed to identify functional groups within the layered materials and modifiers, using KBr pellets (wavenumber range 400–4000 cm^−1^). Thermogravimetric analysis (TGA) was conducted on an SII TG/DTA 6200 system, Nano Technology Inc. Tokyo, Japan, with sample masses below 10 mg, placed in Al_2_O_3_ crucibles under an air atmosphere over a temperature range of 30–950 °C at a heating rate of 10 °C/min [28]. Air was selected to simulate practical service conditions and evaluate thermo-oxidative stability relevant to outdoor PMMA applications. The morphology and microstructure of the samples were examined using polarized optical microscope (POM, OLYMPUS^®^ BX51, Olympus Corporation, Tokyo, Japan), scanning electron microscopy (SEM, JEOL JSM-7600F, JEOL Ltd., Tokyo, Japan) at an accelerating voltage of 10 kV and magnifications ranging from 5 k to 10 k, as well as transmission electron microscopy (TEM, JEOL JEM-1200EX, JEOL Ltd., Tokyo, Japan) operating at 100 kV with a tungsten filament as the electron source (energy spread 0.5~1 eV, operating temperature ~2800 K, and beam current 70 μA), at magnifications between 5 k and 150 k.

Thermal transitions were analyzed using differential scanning calorimetry (DSC, TA Instruments Q10, New Castle, DE, USA) in the temperature range of −50–220 °C at a heating rate of 10 °C/min. The thermo-mechanical behavior was further characterized by dynamic mechanical analysis (DMA, TA Instruments Q800, New Castle, DE, USA) using rectangular specimens of 35 × 12 × 3 mm^3^, measured in the temperature range of −40–80 °C with a heating rate of 5 °C/min [29].

Optical properties were measured using a VARIAN Cary 100 UV-Vis spectrophotometer (Agilent Technologies, Santa Clara, CA, USA) in the wavelength range of 200–800 nm using free-standing films of uniform thickness.

Dynamic light scattering (DLS) measurements were carried out using a Brookhaven Instruments 90 Plus analyzer, Holtsville, NY, USA. A 5 vol% Na_2_HPO_4_ aqueous solution without LDH platelets was used as the solvent blank. A small amount of inorganic filler was dispersed in a 5 vol% Na_2_HPO_4_ aqueous solution (refractive index 1.3345), followed by sonication for 15 min. The suspension was placed in a quartz cuvette, equilibrated for 30 s, and measured three times under identical conditions. The average platelet size and polydispersity index (PDI) were determined from the statistical results.

## 4. Conclusions

This study demonstrates that the multifunctional performance of transparent PMMA/LDH nanocomposites is strongly governed by the coupled effects of LDH platelet size, interlayer modifier chemistry, and dispersion quality. Systematic comparisons among MgAl-LDH and ZnAl-LDH systems revealed that larger and more crystalline LDH platelets generally achieved higher intercalation levels, while optimal thermal and mechanical performance depended primarily on nanoscale dispersion rather than intercalation amount alone. Notably, CDS-modified LDHs exhibited the highest intercalation degrees but poorer dispersion and inferior thermal stability, confirming that high intercalation does not necessarily translate into superior composite performance.

These findings establish a clear structure-property relationship linking LDH crystallinity, gallery accessibility, interfacial compatibility, and composite performance. Well-dispersed SAS-modified LDHs significantly enhanced thermal stability, storage modulus, and UV-shielding capability while maintaining high visible-light transparency. The results further demonstrate that both Mie scattering and aromatic π→π* absorption contribute to UV attenuation, with their relative importance depending on LDH composition and platelet size. This work therefore provides practical design guidelines for developing transparent polymer nanocomposites with simultaneously improved thermal, mechanical, and optical properties.

Beyond PMMA systems, the design principles identified in this study can be extended to a broad range of transparent engineering polymers and optical functional materials. Future studies should explore additional LDH compositions, interlayer chemistries, and hybrid modification strategies to further optimize dispersion, long-term durability, weathering resistance, and UV-shielding efficiency. Such developments may accelerate the use of LDH-based nanofillers in advanced optical components, protective coatings, energy-related devices, and biomedical materials.

## Figures and Tables

**Table 1:** Summary of DLS sizes of LDH under different synthesis conditions.

LDH-Sample	Target Size (nm)	SEM (nm)	DLS (nm)
MgAl-LDH-L-1	L 200	171–254	219.5 (±6.3)
MgAl-LDH-M-1	M 100	63.3–104	101.8 (±4.9)
MgAl-LDH-S-1	S 50	57.3–65.1	67.7 (±3.1)
MgAl-LDH-L-5	L 200	189–214	189.0 (±5.8)
MgAl-LDH-M-5	M 100	51.4–71.8	87.2 (±5.1)
MgAl-LDH-S-5	S 50	38.3–48.8	48.0 (±4.3)
ZnAl-LDH-L-10	L 400	236–360	375.5 (±18)
ZnAl-LDH-M-10	M 300	222–338	297.0 (±18)
ZnAl-LDH-S-10	S 200	159–256	211.2 (±19)

**Table 2:** Interlayer spacing of MgAl-LDH with different modifiers.

MgAl-LDH	(1) 2θ, d_003_	(2) 2θ, d_006_	(3) 2θ, d_009_
MgAl-LDH-L	11.42°, 7.75 Å	22.92°, 3.88Å	34.63°, 2.59 Å
MgAl-L-SAS	6.09°, 14.51 Å	11.87°, 7.45 Å	17.65°, 5.02 Å
MgAl-L-DSMI	3.54°, 24.97 Å	6.72°, 13.15 Å	10.47°, 8.45 Å
MgAl-L-CDS	3.15°, 28.06 Å	6.19°, 14.28 Å	9.37°, 9.44 Å

**Table 3:** Interlayer spacing of ZnAl-LDH with SAS modifiers.

ZnAl-LDH	(1) 2θ, d_003_	(2) 2θ, d_006_	(3) 2θ, d_009_
ZnAl-LDH-L	11.88°, 7.45 Å	23.77°, 3.75 Å	34.89°, 2.57 Å
ZnAl-L-SAS	5.83°, 15.17 Å	11.68°, 7.58 Å	17.47°, 5.08 Å

**Table 4:** Comparison of intercalation amounts for LDHs of different platelet sizes modified with SAS, DMSI, and CDS.

Sample	Intercalation Amount %	Sample	Intercalation Amount %	Sample	Intercalation Amount %
MgAl-S-SAS	46.5 (±1.2)	MgAl-S-DMSI	34.9 (±0.8)	MgAl-S-CDS	92.2 (±0.4)
MgAl-M-SAS	53.2 (±1.5)	MgAl-M-DMSI	45.4 (±1.1)	MgAl-M-CDS	94.0 (±0.2)
MgAl-L-SAS	68.6 (±1.2)	MgAl-L-DMSI	50.0 (±1.6)	MgAl-L-CDS	97.3 (±0.2)
ZnAl-S-SAS	60.1 (±0.9)	ZnAl-M-SAS	62.3 (±1.1)	ZnAl-L-SAS	64.8 (±0.6)

**Table 5:** Functional groups correspond to the absorption peak positions.

Absorption Peak Wavenumber (cm^−1^)	Functional Groups
1. 450 cm^−1^	Mg/Zn–O lattice bending
2. 550 cm^−1^	Al–O lattice bending
3. 750–840 cm^−1^	Aromatic ring substitution vibrations
4. 1050 cm^−1^	Symmetric stretching vibration of SO_3_^−^
5. 1380 cm^−1^	NO_3_^−^ stretching vibration
6. 1450 cm^−1^	Aromatic C=C out-of-plane vibration
7. 1600 cm^−1^	Aromatic C=C out-of-plane vibration
8. 1650 cm^−1^	Stretching vibration of -NH_2_
9. 1720 cm^−1^	Stretching vibration of C=O

**Table 6:** Thermal, mechanical, and optical properties of PMMA and PMMA/LDH nanocomposites at different LDH loadings.

Sample	T_5d_ (°C)	Storage Modulus (MPa)	Visible Transmittance at 550 nm (%)	UVA Transmittance at 320 nm (%)	UVB Transmittance at 280 nm (%)
Pure PMMA	300.4 (±2.3)	397 (±23)	92.1 (±0.5)	88.7 (±0.5)	82.1 (±0.5)
PMMA/MgAl-M-SAS-3 phr	322.5 (±1.4)	612 (±16)	88.8 (±0.6)	81.6 (±0.6)	74.7 (±0.6)
PMMA/MgAl-L-CDS-3 phr	289.5 (±1.3)	376 (±11)	88.5 (±0.5)	76.6 (±0.5)	67.7 (±0.6)
PMMA/ZnAl-M-SAS-1 phr	324.0 (±1.8)	622 (±21)	89.5 (±0.6)	68.8 (±0.3)	61.7 (±0.5)
PMMA/ZnAl-S-SAS-1 phr	318.1 (±1.1)	600 (±27)	86.2 (±0.3)	68.3 (±0.2)	62.4 (±0.3)
PMMA/ZnAl-S-SAS-0.5 phr	303.2 (±0.9)	469 (±29)	88.3 (±0.3)	73.1 (±0.1)	63.6 (±0.2)

## Data Availability

The data supporting this study’s findings are available upon request from the authors.
